# Supplementary material for: Distinct roles for H4 and H2A.Z acetylation in RNA transcription in African trypanosomes
Source: Nat Commun. 2020 Mar 20;11:1498. doi: 10.1038/s41467-020-15274-0 (PMC7083915; doi:10.1038/s41467-020-15274-0)
Supplement: Supplementary file 1 — Supplementary Information [file 41467_2020_15274_MOESM1_ESM.pdf]

Distinct roles for H4 and H2A.Z acetylation in RNA transcription in African trypanosomes

Kraus et al.

Supplementary Information

Table of Contents

Supplementary Figures ***2***

Supplementary Figure 1: N-terminal TY1-tag does not influence targeted H2A.Z deposition. 2

Supplementary Figure 2: Outline of different chromatin isolation approaches. 3

Supplementary Figure 3: TY1-tagged H2A allows co-immunoprecipitation of all other core histones. 5

Supplementary Figure 4: Extended version of Figure 2. 6

Supplementary Figure 5: H2A.Z harbors a long N-terminal domain. 7

Supplementary Figure 6: Extended version of Figure 3. 8

Supplementary Figure 7: MS/MS spectra of newly identified histone phosphorylation marks 10

Supplementary Figure 8: Depletion of HAT1 and HAT2 causes severe growth phenotypes. 11

Supplementary Figure 9: Extended version of Figure 5. 12

Supplementary Figure 10: Lysines of H4 co-immunoprecipitated with TSS-nucleosomes are still acetylated after HAT2 depletion. 13

Supplementary Figure 11: Depletion of HAT1 and HAT2 only affect TSS-specific histone acetylation. 14

Supplementary Figure 12: HAT2 depletion reduces H2A.Z incorporation. 15

Supplementary Figure 13: H2A.Z deposition only changes minimally after HAT1 depletion. 16

Supplementary Figure 14: HAT2 depletion shifts RNA Pol II binding site at TSSs. 17

Supplementary Figure 15: HAT1 depletion reduces RNA Pol II transcript levels. 18

Supplementary Materials and Methods ***19***

Knock down of HAT1 and HAT2 .... **19**

Generation of constructs for inducible knock down of HAT1 and HAT2 19

Generation of HAT1 and HAT2 knock down cell lines 19

TY1-tagging of RNA Pol II subunit *RPB9* **19**

Generation of constructs for endogenous TY1-tagging of both *RPB9* alleles 19

Generation of $^{2xTY/2xTY}$*RBP9* cell lines 20

Statistical analysis of the impact of HAT depletion on histone acetylation levels **20**

Meta-plot generation for binning PTUs **21**

Quantification of western blot using ImageJ **21**

Supplementary References ***21***

Supplementary Figures

Supplementary Figure 1: N-terminal TY1-tag does not influence targeted H2A.Z deposition.

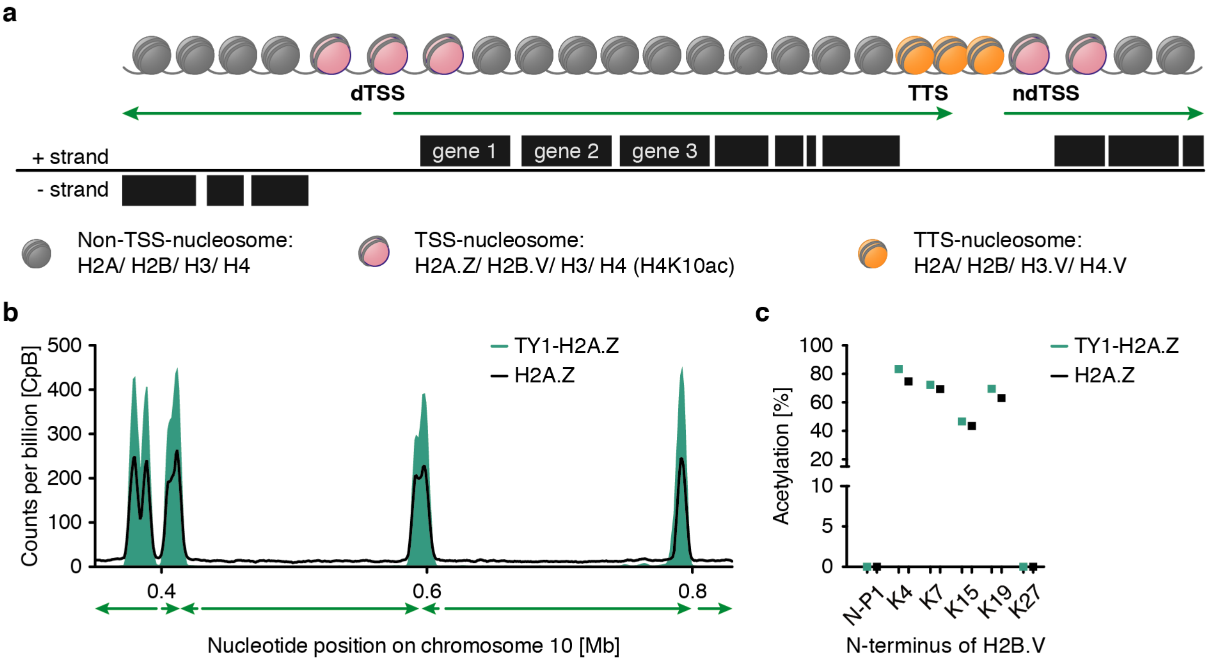

Supplementary Figure 1. N-terminal TY1-tag does not influence targeted H2A.Z deposition. **a** Outline of genome organization in *Trypanosoma brucei*. Genes are arranged as long polycistronic transcription units (PTUs). Divergent transcription start sites (dTSSs) and non-divergent transcription start sites (ndTSSs) are enriched in nucleosomes containing the histone variants H2A.Z and H2B.V (pink). The histone variants H3.V and H4.V replace the canonical histones H3 and H4 in nucleosomes (orange) at transcription termination sites (TTSs). **b** MNase-ChIP-seq data of untagged H2A.Z (black) from wild type (WT) cells (n=1) and of TY1-tagged H2A.Z (n=1) from cells expressing a tagged version of the variant (teal) are shown across a representative region of chromosome 10. The ChIP-seq data are normalized to the total number of reads and are plotted as counts per billion reads [CpB]. **c** Lysine-specific acetylation levels are shown for the N-terminus of H2B.V from TSS-nucleosomes enriched by immunoprecipitation of untagged H2A.Z (black) and TY1-tagged H2A.Z (teal). The acetylation percentages represent the averages of the median values from each of the independent experiments (IP of untagged H2A.Z n=1 and IP of TY1-tagged H2A.Z n=6) determined by FIPQuant. The acetylation percentages [%] of each replicate are plotted in Supplementary Figure 6b (H2B.V panel). The acetylation percentage of K17 could not be determined for TSS-nucleosomes enriched by immunoprecipitation of untagged H2A.Z. Source data are provided in the Source Data File.

Supplementary Figure 2: Outline of different chromatin isolation approaches.

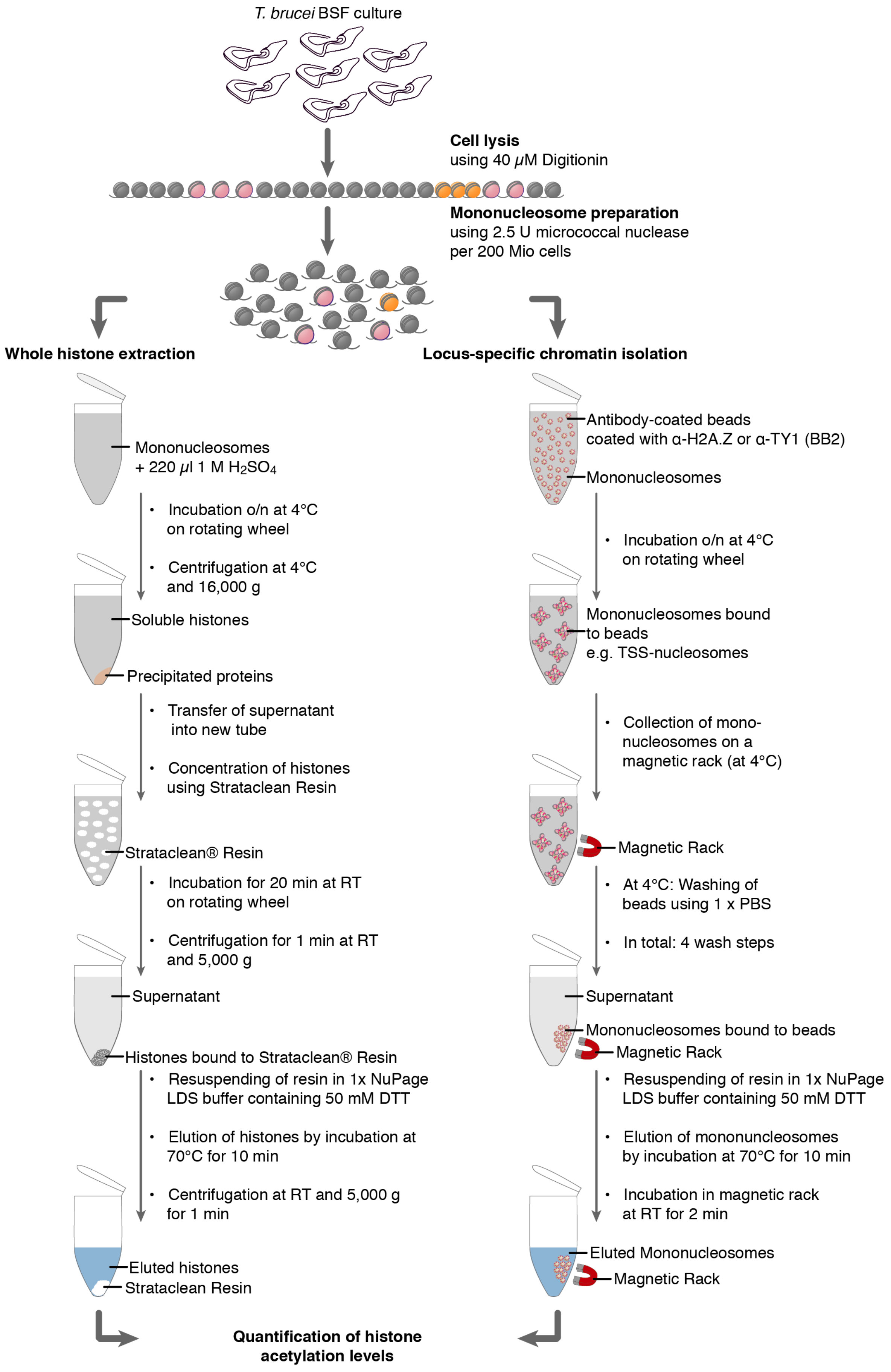

Supplementary Figure 2. Chromatin isolation strategies for genome-wide or locus-specific analyses. Both approaches require the extraction of mononucleosomes as input. Therefore, cells are lysed using digitonin and mononucleosomes are generated by micrococcal nuclease digestion. For genome-wide chromatin analysis (left panel), histones are extracted from the mononucleosomes-containing supernatant by acid extraction, using established protocols [1]. Following concentration using the Strataclean® Resin, histones are eluted from the resin in 1x NuPage LDS buffer containing 50 mM DTT at 70 °C for 10 min. For locus-specific chromatin isolation (right panel), mononucleosomes containing the locus-specific chromatin mark, e.g. H2A.Z, are immunoprecipitated using an antigen-specific antibody, e.g. α-H2A.Z, bound to magnetic beads overnight at 4 °C. Mononucleosomes containing the mark of interest are eluted from the magnetic beads using 1x NuPage LDS buffer containing 50 mM DTT at 70 °C for 10 min. The eluates of both chromatin isolation strategies are used as inputs for quantification of histone acetylation levels.

Supplementary Figure 3: TY1-tagged H2A allows co-immunoprecipitation of all other core histones.

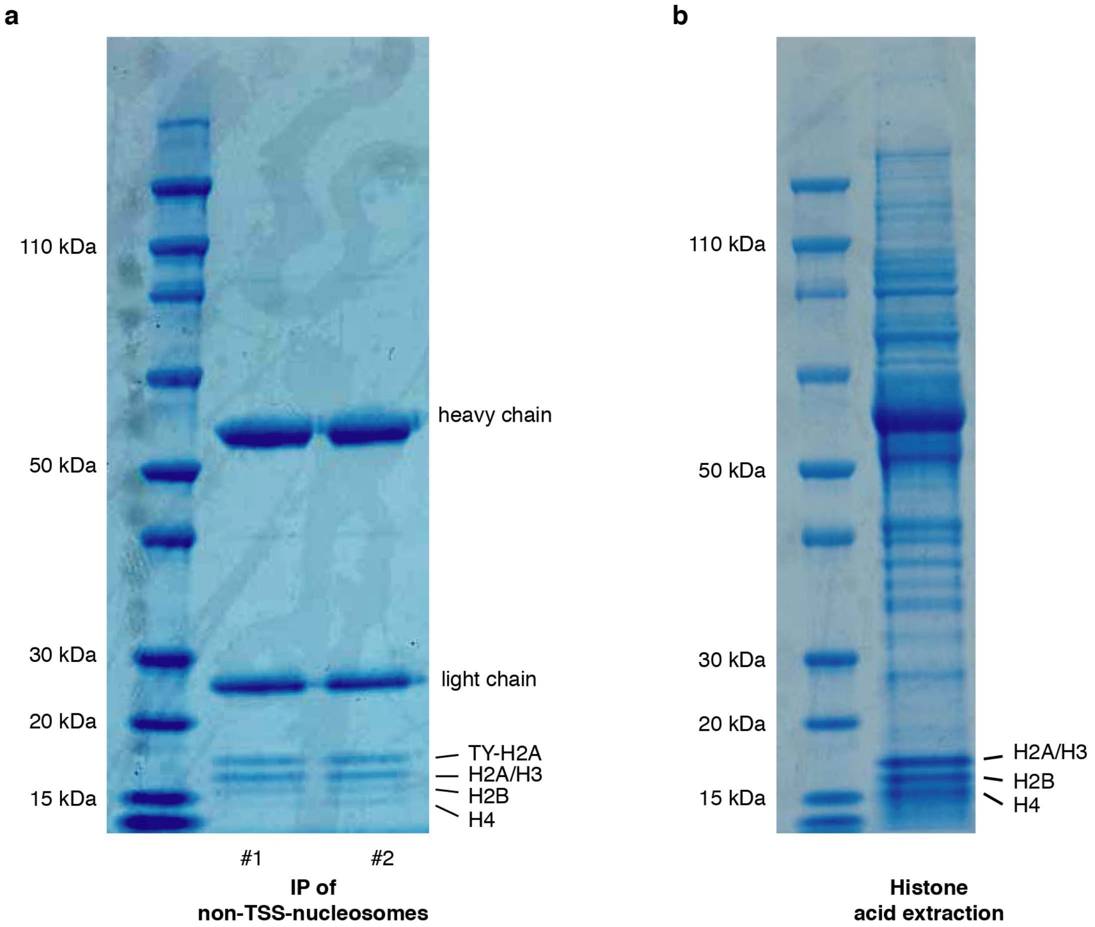

Supplementary Figure 3. TY1-tagged H2A allows co-immunoprecipitation of all other core histones. **a** Coomassie-stained gel of eluate from TY1-H2A-IP (non-TSS-nucleosomes; n=3). **b** Coomassie-stained gel of acid-extracted proteins from wild type cells (n=3).

Supplementary Figure 4: Extended version of Figure 2.

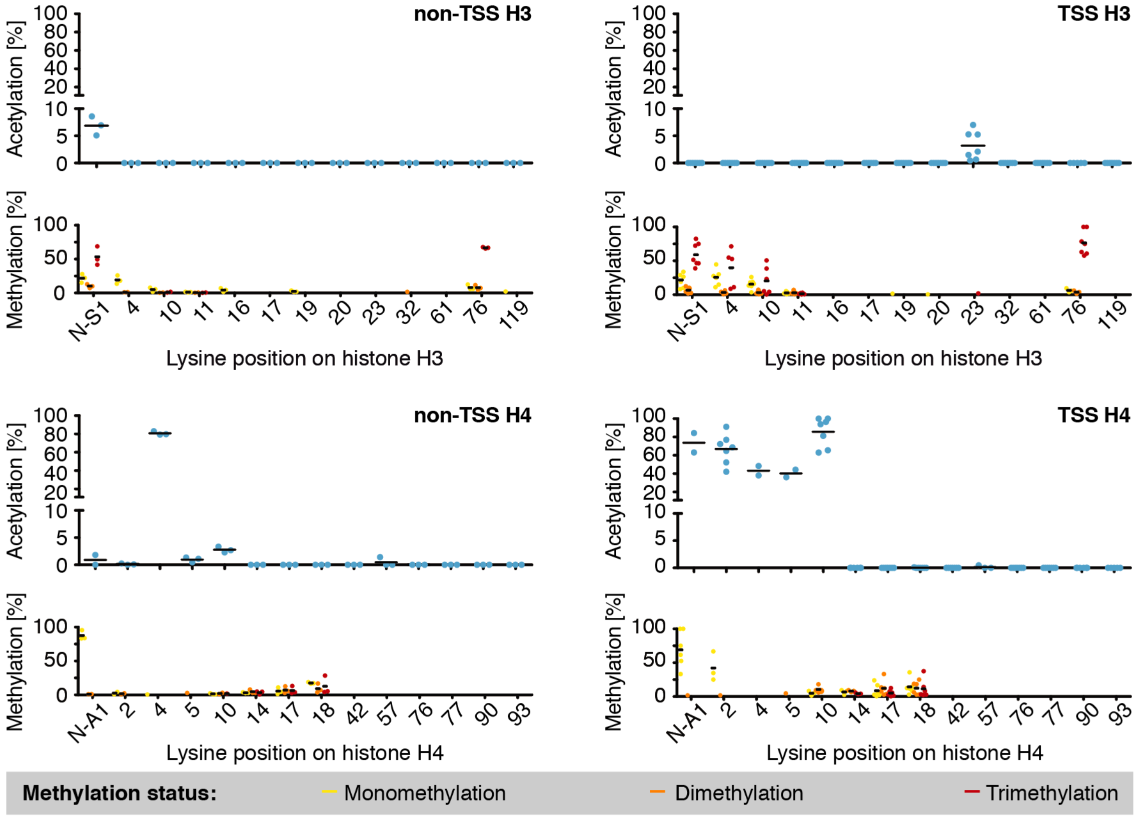

Supplementary Figure 4. Extended version of Figure 2. TSS- and non-TSS-nucleosomes were enriched by immunoprecipitation of TY1-H2A.Z and TY1-H2A-containing nucleosomes, respectively. The plotted acetylation percentages [%] (blue) represent the average percentages of the quantified fragment numbers calculated for each replicate (left panel n=3, right panel n=7). The black bars indicate the average percentages calculated from the median values. The levels of mono- (yellow), di- (orange) and trimethylated (red) spectra identified in each replicate are shown for H3 and H4 from non-TSS- (left panel; n=3) and TSS-nucleosomes (right panel; n=7). The black bars indicate the average percentages of mono-, di- and trimethylated spectra (lower panels). Source data are provided in the Source Data File for Figure 2.

Supplementary Figure 5: H2A.Z harbors a long N-terminal domain.

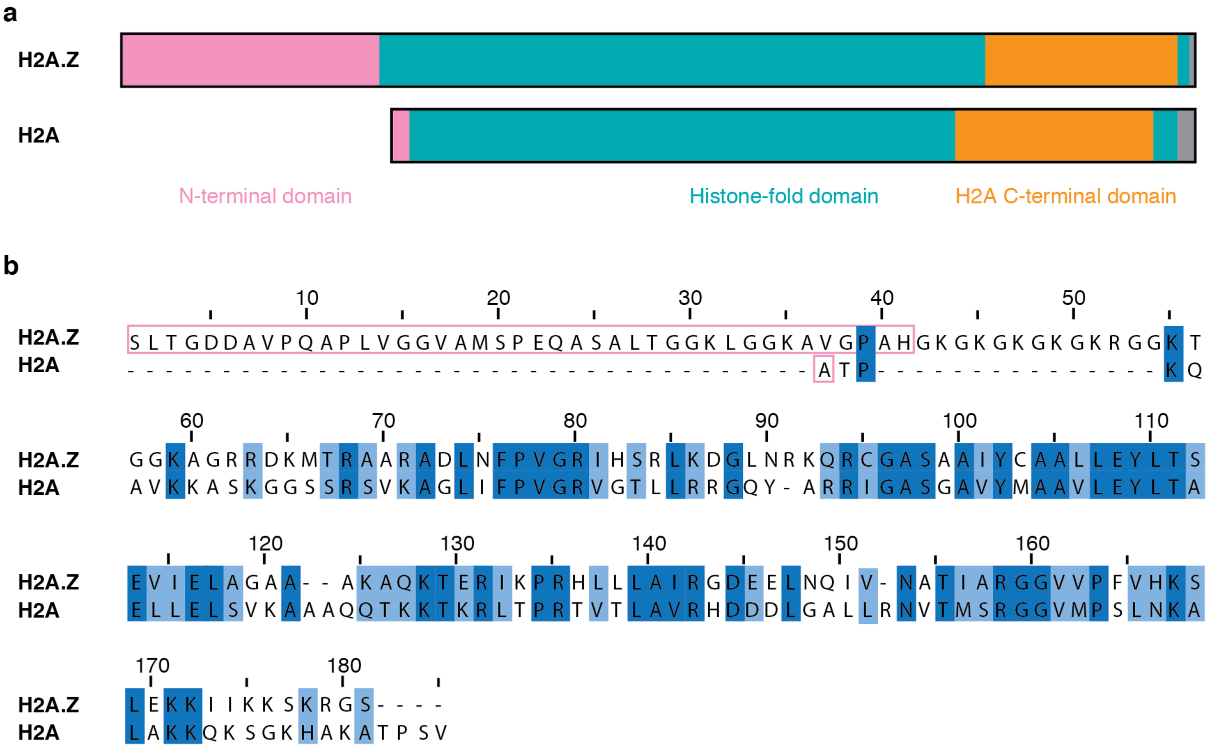

Supplementary Figure 5. H2A.Z contains a long N-terminal domain. **a** Shown is the comparison of the protein length between the histone variant H2A.Z and its canonical counterpart H2A of *T. brucei*. The N-terminus of H2A.Z is 40 aa longer than the N-terminus of H2A (rose box). **b** Alignment of the H2A.Z to the H2A protein sequence shows that the histone-fold and the C-terminal domains share many fully conserved residues (dark blue) and residues with similar properties (light blue). The alignment was obtained using Clustal Omega with standard parameters [2].

Supplementary Figure 6: Extended version of Figure 3.

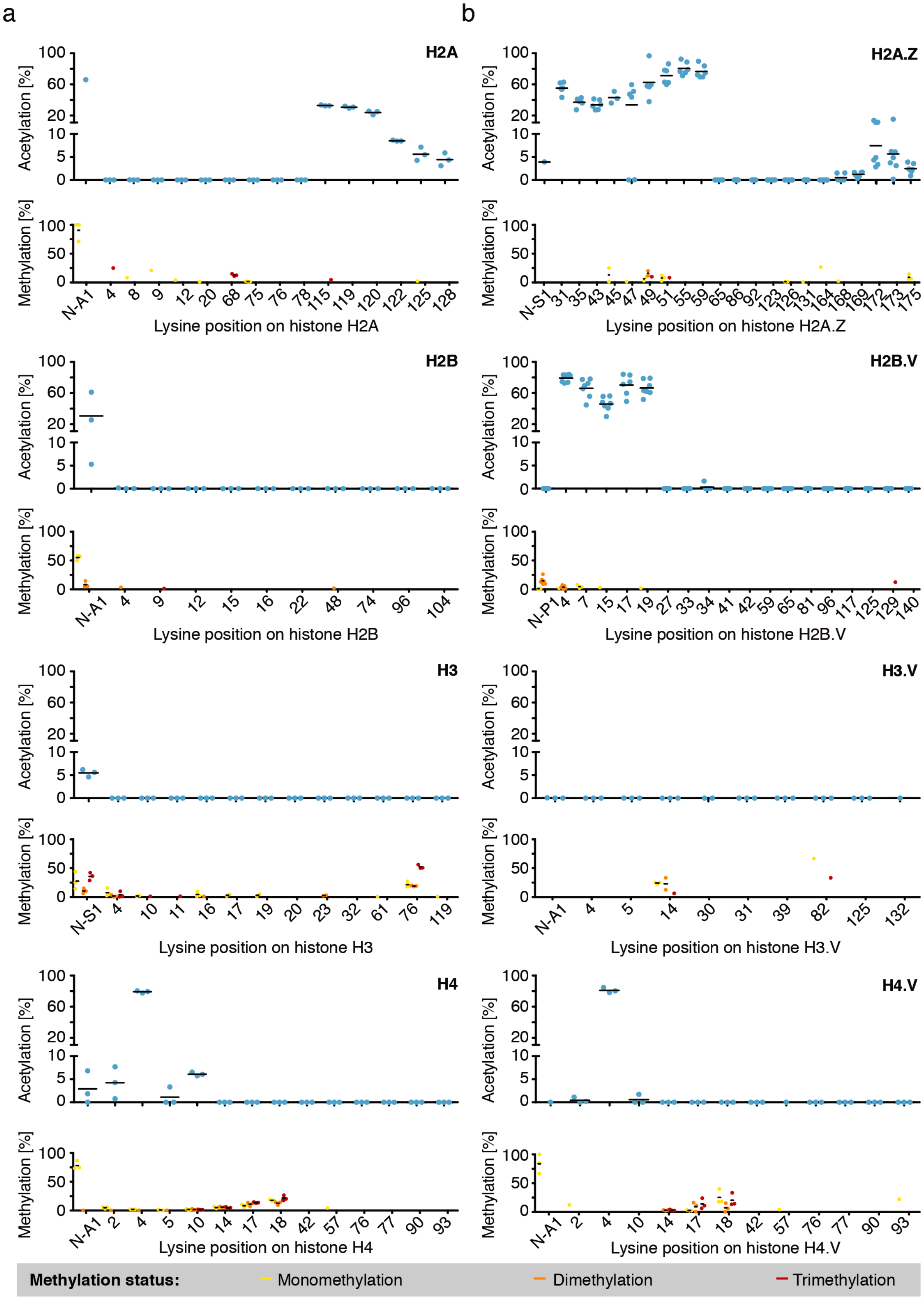

Supplementary Figure 6. Extended version of Figure 3. **a** Lysine-specific acetylation levels (blue) are shown for core histones H2A, H2B, H3 and H4 and were determined by FIPQuant using whole histone extracts of WT cells (upper panels). The plotted acetylation percentages [%] represent the average percentages of the quantified fragment numbers calculated for each replicate (n=3). The black bars indicate the average percentages calculated from the median values. The levels of mono- (yellow), di- (orange) and trimethylated (red) spectra identified in each replicate (n=3) are plotted. The black bars indicate the average percentages of mono-, di- and trimethylated spectra (lower panels). **b** Lysine-specific acetylation levels are shown for histones variants H2A.Z, H2B.V, H3.V and H4.V (blue) and were determined by FIPQuant using whole histone extracts of WT cells (H3.V and H4.V) or immunoprecipitation of TSS-nucleosomes (H2A.Z and H2B.V). The plotted acetylation percentages represent the average percentages of the quantified fragment numbers, calculated for each replicate (H2A.Z and H2B.V: n=7; H3.V and H4.V: n=3). The black bars indicate the average percentages calculated from the median values. The levels of mono- (yellow), di- (orange) and trimethylated (red) spectra identified in each replicate (H2A.Z and H2B.V: n=7; H3.V and H4.V: n=3) are plotted. The black bars indicate the average percentages of mono-, di- and trimethylated spectra (lower panels). Source data are provided in the Source Data File for Figure 3.

Supplementary Figure 7: MS/MS spectra of newly identified histone phosphorylation marks

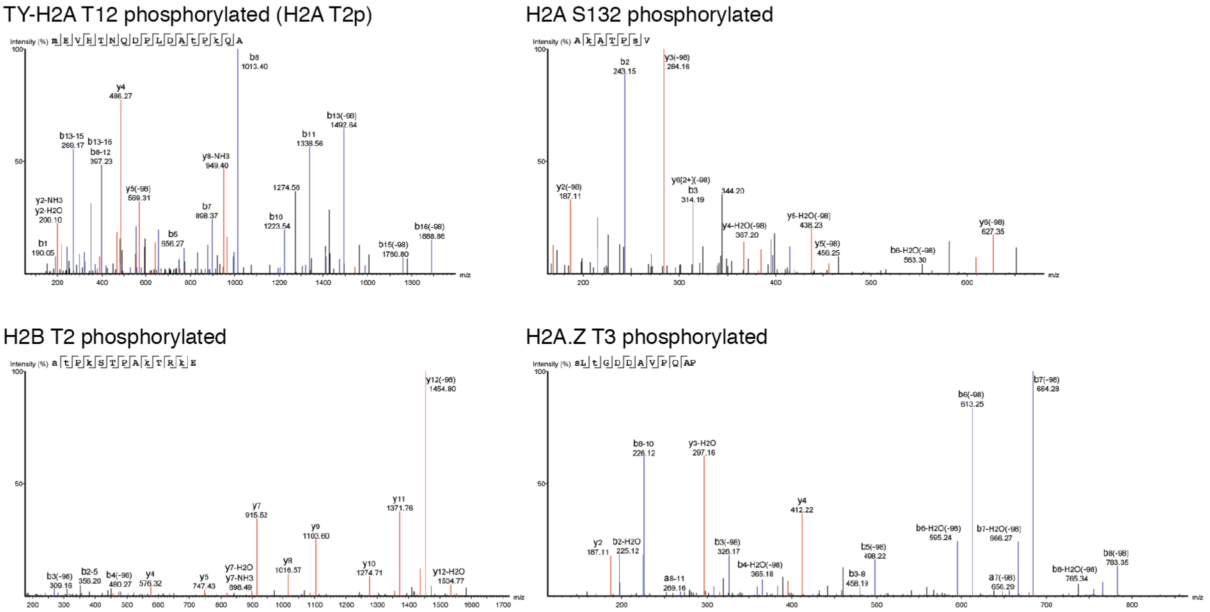

Supplementary Figure 7. MS/MS spectra of identified histone phosphorylation sites. TY1-H2A T12phospho (H2A T2p): HCD Fragment ion spectrum of the peptide A(13C1acK)ATP(pS)V (upper panel; left). H2A S132phospho: HCD Fragment ion spectrum of the peptide A(13C1acK)ATP(pS)V (upper panel; right). H2B T2phospho: HCD Fragment ion spectrum of the peptide (13C1acmeA)(pT)P(13C1acK)STPA(13C1acK)TR(13C1acK)E (lower panel; left). H2A.Z T3phospho: HCD Fragment ion spectrum of the peptide (acS)L(pT)GDDAVPQAP (lower panel; right).

Supplementary Figure 8: Depletion of HAT1 and HAT2 causes severe growth phenotypes.

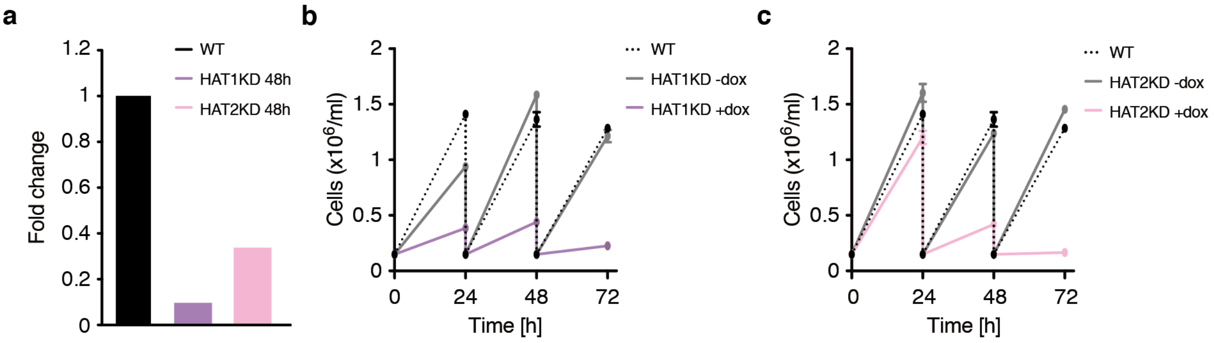

Supplementary Figure 8. Depletion of HAT1 and HAT2 causes severe growth phenotypes. **a** Following depletion of HAT1 (purple; n=3) and HAT2 (rose; n=3) transcript levels were determined by RNA-seq, normalized using external spike-ins and the transcript levels relative to wild type (WT; n=3) cells (black) were calculated using DeSeq2 [3] (Supplementary Data 2). **b** Growth analyses of WT cells (black; dotted line; n=3) and 2T1 cells, induced for HAT1 depletion (+dox; purple; n=3) or not induced (-dox; grey; n=3), are shown over a time period of 72 h. Cells were diluted to 0.15 x 10^6 cells per ml every 24 h. Plotted are the averages of the cell concentrations measured for each replicate (n=3). Error bars indicate standard deviations. **c** Growth analyses of WT cells (black; dotted line) and 2T1 cells, induced for HAT2 depletion (+dox; rose) or not induced (-dox; grey), are shown over a time period of 72 h. Cells were diluted to 0.15 x 10^6 cells per ml every 24 h. Plotted are the averages of the cell concentrations measured for each replicate (n=3). Error bars indicate standard deviations.

Supplementary Figure 9: Extended version of Figure 5.

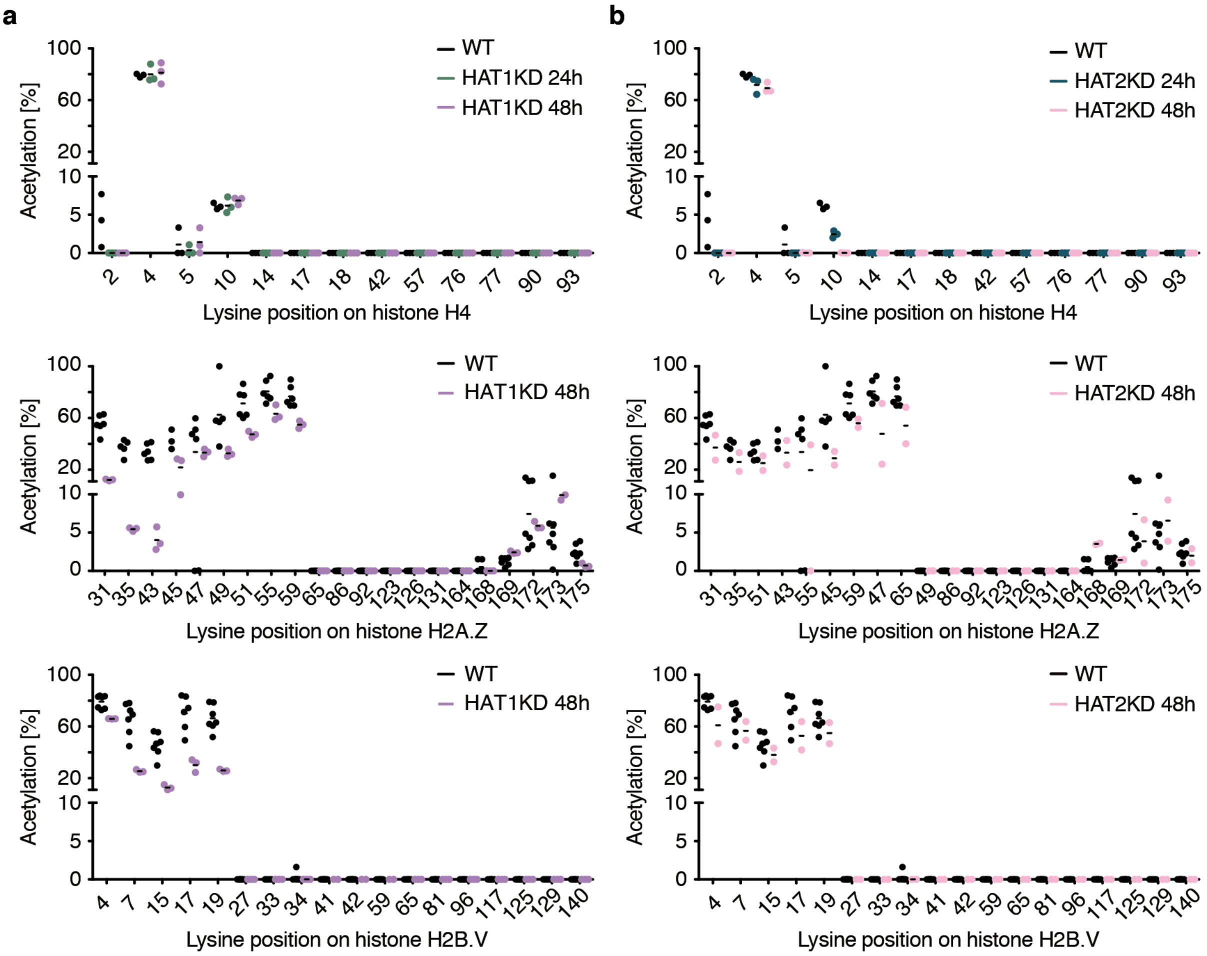

Supplementary Figure 9. Extended version of Figure 5. **a** Lysine-specific acetylation levels for histone H4, H2A.Z and H2B.V are shown for wild type (WT) cells and after depletion of HAT1. H4 acetyl marks were quantified by FIPQuant using histones extracted from WT cells (black; n=3) and from 2T1 cells, which were depleted of HAT1 for 24 h (green; n=3) or 48 h (purple; n=3). H2A.Z and H2B.V acetyl marks were quantified using histones from immunoprecipitated TSS-nucleosomes of WT cells (black; n=7) and 2T1 cells, depleted for HAT1 for 48 h (purple; n=3). The plotted acetylation percentages [%] represent the average percentages of the quantified fragment numbers calculated for each replicate. The black bars indicate the average percentages calculated from the median values. **b** Lysine-specific acetylation levels for histone H4, H2A.Z and H2B.V are shown for WT cells and after depletion of HAT2. H4 acetyl marks were quantified by FIPQuant using histones extracted from WT cells (black; n=3) and from 2T1 cells, which were depleted of HAT2 for 24 h (blue; n=3) or 48 h (rose; n=3). H2A.Z and H2B.V acetyl marks were quantified using histone from immunoprecipitated TSS-nucleosomes of WT cells (black; n=7) and 2T1 cells, depleted for HAT2 for 48 h (rose; n=2). The plotted acetylation percentages represent the average percentages of the quantified fragment numbers calculated for each replicate. The black bars indicate the average percentages calculated from the median values. Source data are provided in the Source Data File for Figure 5.

Supplementary Figure 10: Lysines of H4 co-immunoprecipitated with TSS-nucleosomes are still acetylated after HAT2 depletion.

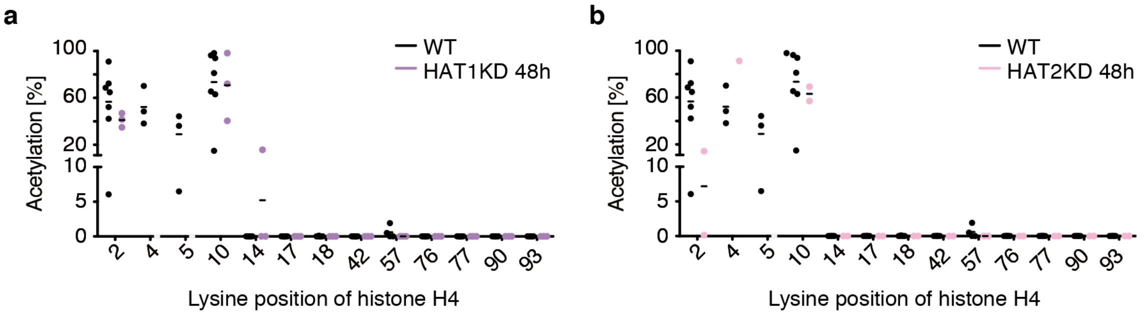

Supplementary Figure 10. Lysines of H4 co-immunoprecipitated with TSS-nucleosomes are still acetylated after HAT2 depletion. **a** Lysine-specific acetylation levels for histone H4 at TSS-nucleosomes are shown for wild type (WT) cells and after depletion of HAT1. H4 acetyl marks were quantified using histones from immunoprecipitated TSS-nucleosomes of WT cells (black; n=7) and 2T1 cells, depleted for HAT1 for 48 h (purple; n=3). The plotted acetylation percentages represent the average percentages of the quantified fragment numbers, calculated for each replicate. The black bars indicate the average percentages calculated from the median values. **b** Lysine-specific acetylation levels for histone H4 at TSS-nucleosomes are shown for WT cells and after depletion of HAT2. H4 acetyl marks were quantified using histones from immunoprecipitated TSS-nucleosomes of WT cells (black; n=7) and 2T1 cells, depleted for HAT2 for 48 h (rose; n=2). The plotted acetylation percentages represent the average percentages of the quantified fragment numbers, calculated for each replicate. The black bars indicate the average percentages calculated from the median values. Source data are provided in the Source Data File.

Supplementary Figure 11: Depletion of HAT1 and HAT2 only affect TSS-specific histone acetylation.

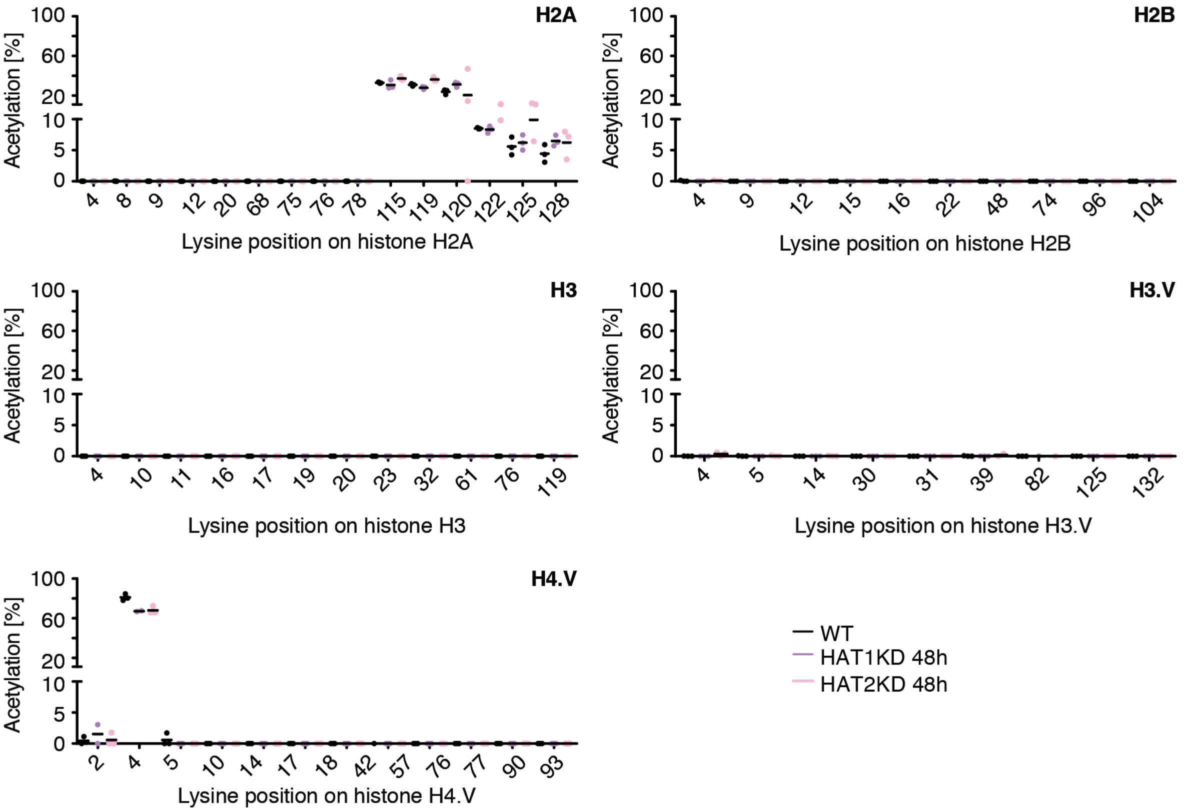

Supplementary Figure 11. Depletion of HAT1 and HAT2 only affects TSS-specific histone acetylation. Lysine-specific acetylation levels for histone H2A, H2B, H3, H3.V and H4.V are shown for wild type (WT; n=3) cells and after depletion of HAT1 (purple; n=3) and HAT2 (rose; n=3). Acetyl marks were quantified by FIPQuant using histones extracted from WT cells (black) and from 2T1 cells, which were depleted of HAT1 (purple) or HAT2 (rose) for 48 h. The plotted acetylation percentages represent the average percentages of the quantified fragment numbers, calculated for each replicate. The black bars indicate the average percentages calculated from the median values. Source data are provided in the Source Data File.

Supplementary Figure 12: HAT2 depletion reduces H2A.Z incorporation.

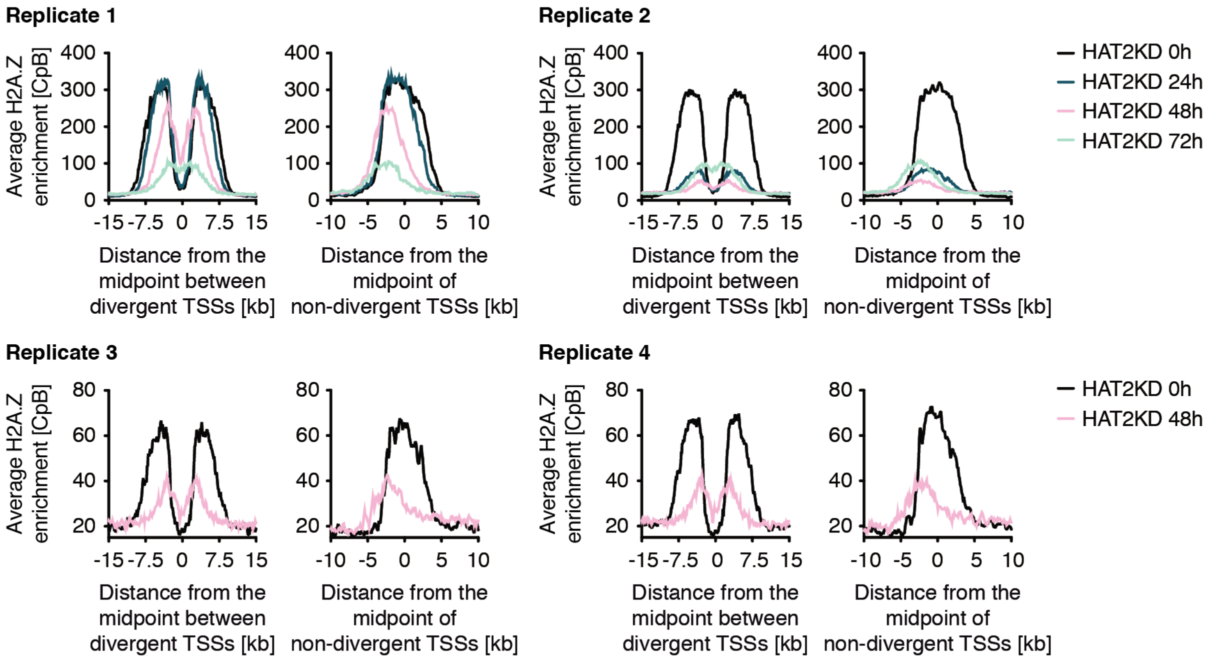

Supplementary Figure 12. HAT2 depletion reduces H2A.Z incorporation. MNase-ChIP-seq datasets of histone variant H2A.Z after 0 h (black; n=4), 24 h (blue; n=2), 48 h (rose; n=4), and 72 h (light green; n=2) HAT2 depletion are averaged across divergent (n=37) and non-divergent (n=49) TSSs. The ChIP-seq data are normalized to the total number of reads and plotted as counts per billion reads [CpB]. The data of replicate 2 after 48 h of HAT2 depletion are also shown in Fig. 6. Source data are provided in the Source Data File.

Supplementary Figure 13: H2A.Z deposition only changes minimally after HAT1 depletion.

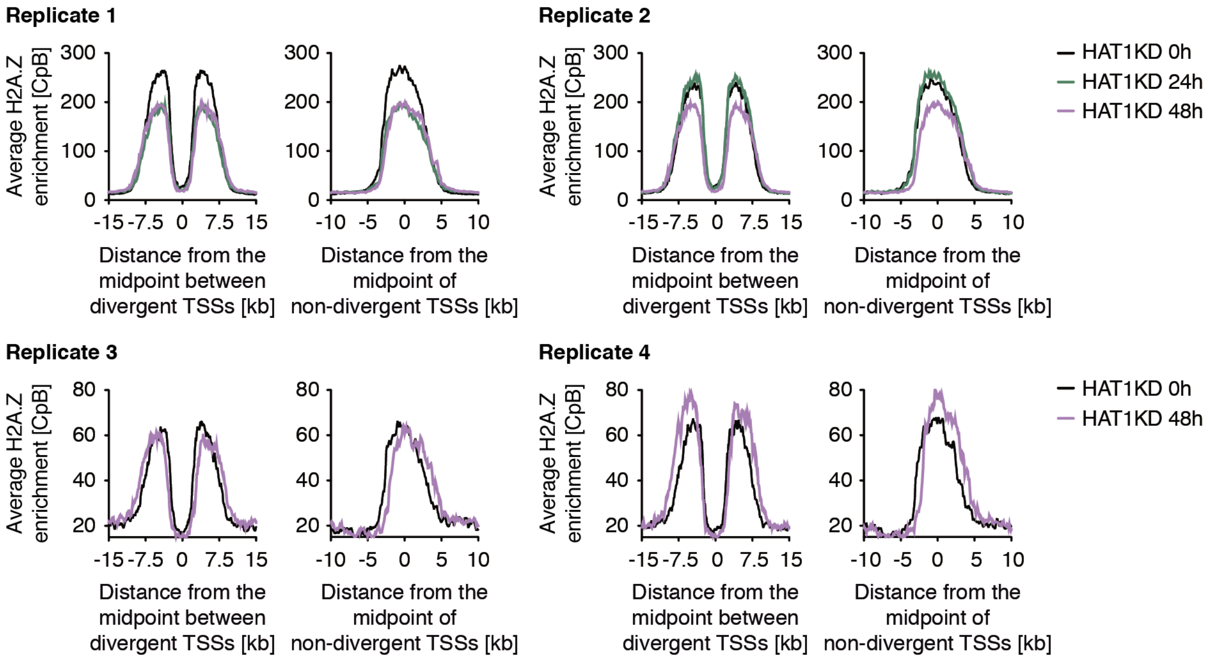

Supplementary Figure 13. H2A.Z deposition only changes minimally after HAT1 depletion. MNase-ChIP-seq datasets of histone variant H2A.Z after 0 h (black; n=4), 24 h (green; n=2), and 48 h (purple; n=4) HAT1 depletion are averaged across divergent (n=37) and non-divergent (n=49) TSSs. The ChIP-seq data are normalized to the total number of reads and plotted as counts per billion reads [CpB]. The data of replicate 1 after 48 h of HAT1 depletion are also shown in Fig. 6. Source data are provided in the Source Data File.

Supplementary Figure 14: HAT2 depletion shifts RNA Pol II binding site at TSSs.

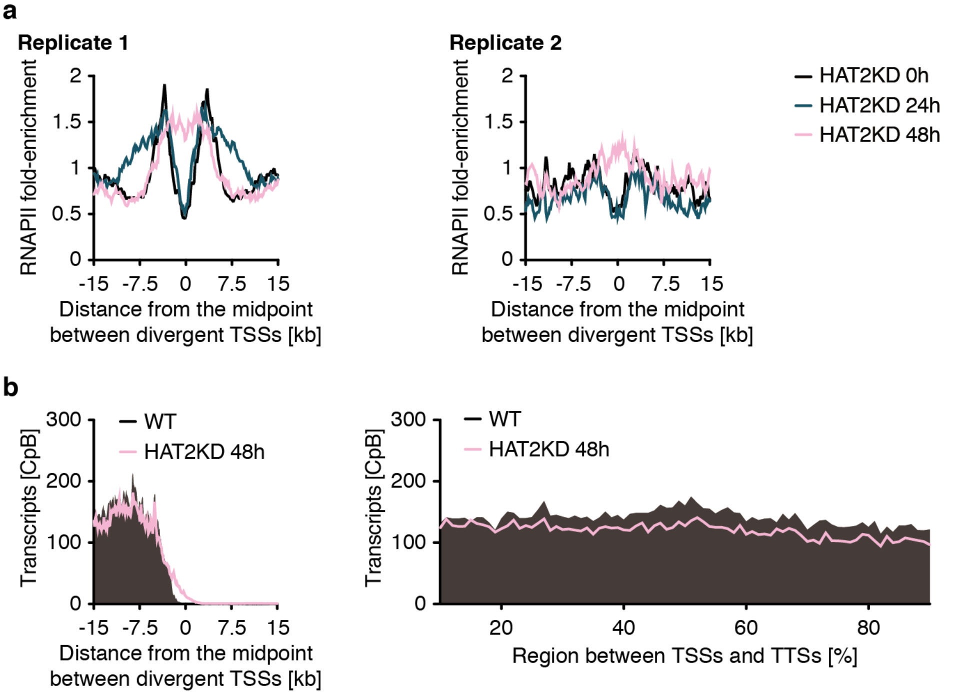

Supplementary Figure 14. HAT2 depletion shifts RNA Pol II binding site at TSSs. **a** ChIP-seq datasets of TY1-tagged RPB9 (RNAPII = RNA Pol II) after 0 h (black; n=2), 24 h (green; n=2), and 48 h (rose; n=2) of HAT2 depletion are averaged across divergent (n=37) TSSs. The data of replicate 1 (left panel) after 48 h of HAT2 depletion are also shown in Fig.7a. **b** RNA-seq data showing transcripts derived from the -strand before (black) and after 48 h HAT2 depletion (rose) are averaged across divergent TSSs (n=37) located on the -strand (left panel). RNA-seq data showing transcripts derived from the +strand before (black) and after 48 h HAT2 depletion (rose) are shown across regions between TSSs and TTSs on the +strand (n=109; right panel). The data are normalized to a spike-in control to account for differences in total RNA levels per cell after HAT2 depletion and plotted as counts per billion reads [CpB]. Shown is the average from three RNA-seq experiments normalized to a spike-in control to account for differences in total RNA level per cell (for normalization factors see Supplementary Data 5). Source data are provided in the Source Data File.

Supplementary Figure 15: HAT1 depletion reduces RNA Pol II transcript levels.

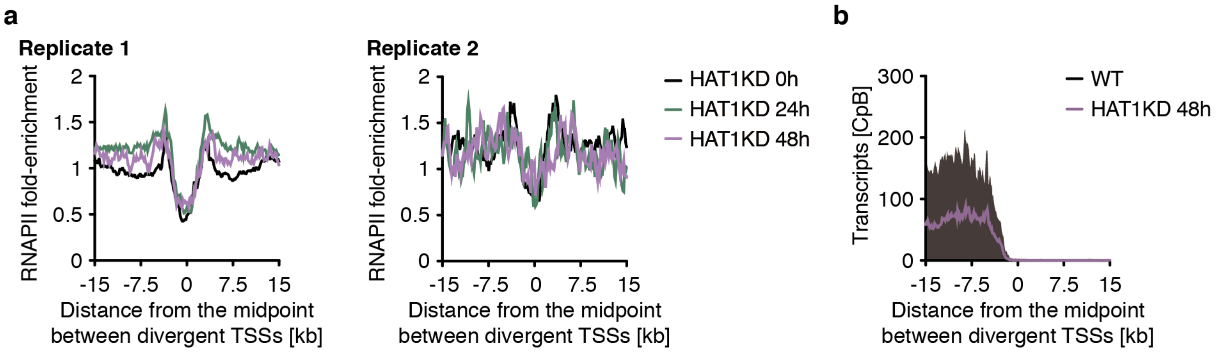

Supplementary Figure 15. HAT1 depletion reduces RNA Pol II transcript levels. **a** ChIP-seq datasets of TY1-tagged RPB9 (RNAPII = RNA Pol II) after 0 h (black; n=2), 24 h (green; n=2), and 48 h (purple; n=2) of HAT1 depletion are averaged across divergent (n=37) TSSs. The data of replicate 1 (left panel) after 48 h of HAT1 depletion are also shown in Fig. 8a. **b** RNA-seq data showing transcripts derived from the -strand before (black) and after 48 h HAT1 depletion (purple) are averaged across divergent TSSs (n=37) located on the -strand. The data are normalized to a spike-in control to account for differences in total RNA levels per cell after HAT1 depletion and plotted as counts per billion reads [CpB]. Shown are the averages from three RNA-seq experiments normalized to a spike-in control to account for differences in total RNA levels per cell (for normalization factors see Supplementary Data 5). Source data are provided in the Source Data File.

Supplementary Materials and Methods

Knock down of HAT1 and HAT2

Generation of constructs for inducible knock down of HAT1 and HAT2

The constructs for inducible knock down of HAT1 and HAT2 originate from pRPa$_{iSL}$ [4], which allows generation of stem-loop RNAs for efficient RNA interference. Oligos for amplifying the targeting regions were ordered, for details see [5]. The HAT1-targeting region (amplified with H1R5SL-GATCGGGCCCGGTACCCCCTTTTCGGAACATGAAGA- and H1R3SL-GATCTCTAGAGGATCCAGTGTGACGCAGTGACTT- from WT gDNA) was digested with KpnI/BamHI to generate the sense targeting region and with XbaI/ApaI to generate the antisense targeting region. To generate pRPa$_{iSL}$_HAT1_KD, the sense targeting region was inserted into pRPa$_{iSL}$ and the antisense targeting region was inserted into the generated pRPa$_{iSL}$_HAT1_sense. The construct pRPa$_{iSL}$_HAT2_KD was generated as described above using the HAT2-targeting region (amplified with H2R5SL-GATCGGGCCCGGTACCACTCACAGACTTGGGAGCA - and H2R3SL-GATCTCTAGAGGATCCTCATGTCATGTGCCCACTTT - from WT gDNA). Before transfection of the cells, pRPa$_{iSL}$_HAT1_KD and pRPa$_{iSL}$_HAT2_KD were digested with AscI.

Generation of HAT1 and HAT2 knock down cell lines

The cell lines for inducible knock down of HAT1 and HAT2 derive from the 2T1 cell line, in which the knock down construct integrates into a PUR-tagged rRNA locus for stable transfection efficiency [4,6]. To generate 2T1_HAT1_KD, the AscI-linearized pRPaiSL_HAT1_KD was transfected into 2T1 cells using a Nucleofector (Amaxa) using an established protocol [7]. 2T1_HAT2_KD cells lines were generated using AscI-linearized pRPaiSL_HAT2_KD. Hygromycin and phleomycin were added to the culture medium to select for successful transfectants. Successful integration of the knock down constructs was validated by PCR (CGCGTCTGCTGCTCCATACAAG and TCAAGCTCTAAATCGGGGGC) on gDNA purified from 2T1_HAT1_KD and 2T1_HAT2_KD cells, respectively. To evaluate knock down efficiencies, growth rates were monitored for 96 h and the transcript levels of HAT1 (CAGAACGCGGAAGGAGAGTT and GCGAGAAGTACCACGTCTCC) and HAT2 (CCCACCACAGTGCGATACTT and TGCGTCGCTTAAAGGTGCTA) were analyzed at different time points by quantitative real-time PCR.

TY1-tagging of RNA Pol II subunit *RPB9*

Generation of constructs for endogenous TY1-tagging of both *RPB9* alleles

The tagging construct pPOTv3-BSD-2xTY originates from pPOTv3 (a precursor from the pPOT-constructs [8]). The YFP gene was exchanged by two succeeding, codon-optimized TY1-tags, with the first one containing the ATG, generated by annealing of two oligonucleotides (AGCTTATGGAGGTGCACACGAACCAGGACCCGCTGGACGCCGAGGTGCACACG AACCAGGACCCGCTGGACGGATCCGGATCAGGATCTGGATCAGGATCGGGTAGT GAGCT and CACTACCCGATCCTGATCCAGATCCTGATCCGGATCCGTCAAGTGGATCTTGGTT AGTATGGACCTCGGCGTCAAGTGGGTCCTGGTTAGTATGGACCTCCATA) using the restriction sites HindIII and SacI.

The cloning reactions for generation of constructs for endogenous TY1-tagging of both *RPB9* alleles were performed using InFusion® HD Cloning Plus CE reagents (cat no. 638916, Takara Bio) according to the manufacturer's instructions. To generate the construct pPOTv3-BSD-2xTY_RPB9 for N-terminal 2xTY1-tagging of the first allele of *RPB9*, the upstream region of *RPB9* (amplified with CTATAGGGCGAATTGGGCCCTGTGTCAACCAACGGCGATA and GCATTATACGCGGCCGCGGTGGTTTATTCCACCTTAGCTTCG from WT gDNA) was integrated into the ApaI/NotI-digested pPOTv3-BSD-2xTY. The downstream region, homologous to the 5'end of RPB9 CDS excluding the ATG (amplified with CAGGATCGGGTAGTGAGCTCGAGTCCACTTTGACGCAG and GCTTTTTCATGCTAGCGTTCACGAAGCATGTGACTTC from WT gDNA) was inserted using SacI/NheI-digested pPOTv3-BSD-2xTY_RPB9_UR. To generate the construct pPOTv3-NEO-2xTY_RPB9 for N-terminal 2xTY1-tagging of the second allele of *RPB9*, the resistance cassette *BSD* was exchanged by a *NEO* cassette (amplified with GCAACGAAGCTTGTTAACATGATTGAACAAGATGGATTGCAC and TGGGCAGGATCCGGATCAGAAGAACTCGTCAAGAAGGC from pyrFE-NEO [9]) using the restriction sites MluI/NotI. Before transfection of the cells, the tagging constructs pPOTv3-BSD-2xTY_RPB9 and pPOTv3-NEO-2xTY_RPB9 were linearized using ApaI/NheI.

Generation of $^{2xTY/2xTY}$*RBP9* cell lines

The cell lines containing $^{2xTY/2xTY}$*RBP9* derive from the 2T1 cell line [4,6]. For tagging the first allele, ApaI/NheI-linearized pPOTv3-BSD-2xTY_RPB9 was transfected into 2T1 cells using a Nucleofector (Amaxa) using an established protocol [7]. Integration of the tagging construct was verified by PCR (TGCTAGCGCCTTATCACCAC and CACAAGGTCCCCCAGTAAAA; GCATCTTCACTGGTGTCAATGTA and CAAAGGTAGGCGGTGGGG) on gDNA purified from 2T1_$^{2xTY/x}$RBP9 cells. For tagging the second allele, ApaI/NheI-linearized pPOTv3-NEO-2xTY_RPB9 was transfected into 2T1_$^{2xTY/x}$RBP9 cells using a Nucleofector (Amaxa). Integration of the tagging construct was verified by PCR (TGCTAGCGCCTTATCACCAC and AATATCACGGGTAGCCAACG; TGAATGAACTGCAGGACGAG and CAAAGGTAGGCGGTGGGG) on gDNA purified from 2T1_$^{2xTY/2xTY}$RBP9 cells. Tagging was further evaluated by western blot analysis. 2T1_$^{2xTY/2xTY}$RBP9 were cultivated adding puromycin, phleomycin, neomycin and blasticidin to the medium.
2T1_$^{2xTY/x}$RBP9_HAT1_KD and 2T1_$^{2xTY/x}$RBP9_HAT2_KD cells lines were generated using AscI-linearized pRPa$_{iSL}$_HAT1_KD and pRPa$_{iSL}$_HAT2_KD, respectively, as described above. 2T1_$^{2xTY/x}$RBP9_HAT1_KD and 2T1_$^{2xTY/x}$RBP9_HAT2_KD cells were cultivated adding hygromycin, phleomycin, neomycin and blasticidin to the medium. Integration of the knock down constructs was analyzed by PCR (CGCGTCTGCTGCTCCATACAAG and TCAAGCTCTAAATCGGGGGC) on gDNA purified from 2T1_$^{2xTY/x}$RBP9_HAT1_KD and 2T1_$^{2xTY/x}$RBP9_HAT2_KD cells, respectively. To evaluate knock down efficiencies, growth rates were monitored for 96 h and the transcript levels of HAT1 (CAGAACGCGGAAGGAGAGTT and GCGAGAAGTACCACGTCTCC) and HAT2 (CCCACCACAGTGCGATACTT and TGCGTCGCTTAAAGGTGCTA) were analyzed at different time points by quantitative real-time PCR.

Statistical analysis of the impact of HAT depletion on histone acetylation levels

Statistical analyses of the impact of HAT depletion on histone acetylation levels were performed using GraphPad Prism version 7.0c. We applied a multiple t-test between the

different conditions, with three replicates per condition. Individual p-values for each lysine position were computed using the two-way ANOVA approach recommended for comparing samples from different conditions https://www.graphpad.com/guides/prism/7/statistics/index.htm?stat_options_for_multiple_t_tests.htm. The statistical significance was determined using the Holm-Sidak method [10,11] to correct for multiple comparison. Adjusted p-values < 0.05 were defined as 'statistically significant' and marked with asterisk in the relevant figures (Supplementary Data 7).

Meta-plot generation for binning PTUs

To be able to average the sequencing coverage data for multiple polycistronic transcription units with different length, COVERnant's subcommand *extract* was extended by the optional argument *--meta_plots*, which calculates the coverage as a fraction relative to the length (https://github.com/bgbrink/COVERnant).

Quantification of western blot using ImageJ

Quantification of the western blot was performed as described by Hossein Davarinejad (Quantifications of Western Blots with ImageJ; http://www.yorku.ca/yisheng/Internal/Protocols/ImageJ.pdf). ImageJ [12] was downloaded from https://imagej.nih.gov/ij/download.html. Western blot quantification was performed under the measurement criteria "Grey Mean Value". For H3 and H2A.Z individual regions of interests (ROI) were selected using the *rectangle* tool. The H3 signal and the H3 background were measured for each lane using the H3 ROI. The H2A.Z signal and the H2A.Z background for each lane using the H2A.Z ROI. The inverted value was calculated for each protein and background value by subtracting the measured value from 255. For calculating the net protein value, the inverted background value was subtracted from the inverted protein value. For relative quantification, the ratio of the net H2A.Z value over the net H3 value (loading control) was calculated. The H2A.Z/H3 ratios from the HAT2 depleted chromatin fractions were compared to the H2A.Z/H3 ratio from the 2T1 chromatin fraction. To quantify the levels of chromatin-bound TY1-RPB9, the calculation was performed as described above for H2A.Z using the levels of H3 as loading control.

Supplementary References

1. Shechter, D., Dormann, H. L., Allis, C. D. & Hake, S. B. Extraction, purification and analysis of histones. *Nat Protoc* **2**, 1445-1457 (2007).
2. Sievers, F. et al. Fast, scalable generation of high-quality protein multiple sequence alignments using Clustal Omega. *Mol Syst Biol* **7**, 539 (2011).
3. Love, M. I., Huber, W. & Anders, S. Moderated estimation of fold change and dispersion for RNA-seq data with DESeq2. *Genome Biol* **15**, 550 (2014).
4. Alsford, S. & Horn, D. Single-locus targeting constructs for reliable regulated RNAi and transgene expression in *Trypanosoma brucei. Mol. Biochem. Parasitol.* **161**, 76-79 (2008).
5. Kawahara, T. et al. Two essential MYST-family proteins display distinct roles in histone H4K10 acetylation and telomeric silencing in trypanosomes. *Mol. Microbiol.* **69**, 1054-1068 (2008).
6. Alsford, S., Kawahara, T., Glover, L. & Horn, D. Tagging a T. brucei RRNA locus improves stable transfection efficiency and circumvents inducible expression position effects. *Mol Biochem Parasitol Molecular and biochemical parasitology* **144**, 142-148 (2005).

7. Scahill, M. D., Pastar, I. & Cross, G. A. M. CRE recombinase-based positive-negative selection systems for genetic manipulation in *Trypanosoma brucei. Mol. Biochem. Parasitol.* **157**, 73-82 (2008).
8. Dean, S. et al. A toolkit enabling efficient, scalable and reproducible gene tagging in trypanosomatids. *Open Biol* **5**, 140197 (2015).
9. Kim, H. S., Li, Z., Boothroyd, C. & Cross, G. A. Strategies to construct null and conditional null Trypanosoma brucei mutants using Cre-recombinase and loxP. *Mol Biochem Parasitol* **191**, 16-19 (2013).
10. RYAN, T. A. Significance tests for multiple comparison of proportions, variances, and other statistics. *Psychol Bull* **57**, 318-328 (1960).
11. Holm, S. A Simple Sequentially Rejective Multiple Test Procedure. *Scandinavian Journal of Statistics* **6**, 65-70 (1979).
12. Schneider, C. A., Rasband, W. S. & Eliceiri, K. W. NIH Image to ImageJ: 25 years of image analysis. *Nat Methods* **9**, 671-675 (2012).
