# Supplementary material for: Distinct roles for H4 and H2A.Z acetylation in RNA transcription in African trypanosomes
Source: Nat Commun. 2020 Mar 20;11:1498. doi: 10.1038/s41467-020-15274-0 (PMC7083915; doi:10.1038/s41467-020-15274-0)
Supplement: Supplementary file 3 — Reporting Summary [file 41467_2020_15274_MOESM3_ESM.pdf]

nature research

Corresponding author(s): T. Nicolai Siegel

Last updated by author(s): Feb 24, 2020

Reporting Summary

Nature Research wishes to improve the reproducibility of the work that we publish. This form provides structure for consistency and transparency in reporting. For further information on Nature Research policies, seeAuthors & Referees and theEditorial Policy Checklist .

Statistics

For all statistical analyses, confirm that the following items are present in the figure legend, table legend, main text, or Methods section.

n/a	Confirmed	
☐	☒	The exact sample size (*n*) for each experimental group/condition, given as a discrete number and unit of measurement
☐	☒	A statement on whether measurements were taken from distinct samples or whether the same sample was measured repeatedly
☐	☒	The statistical test(s) used AND whether they are one- or two-sided<br>*Only common tests should be described solely by name; describe more complex techniques in the Methods section.*
☒	☐	A description of all covariates tested
☒	☐	A description of any assumptions or corrections, such as tests of normality and adjustment for multiple comparisons
☐	☒	A full description of the statistical parameters including central tendency (e.g. means) or other basic estimates (e.g. regression coefficient) AND variation (e.g. standard deviation) or associated estimates of uncertainty (e.g. confidence intervals)
☒	☐	For null hypothesis testing, the test statistic (e.g. *F*, *t*, *r*) with confidence intervals, effect sizes, degrees of freedom and *P* value noted<br>*Give P values as exact values whenever suitable.*
☒	☐	For Bayesian analysis, information on the choice of priors and Markov chain Monte Carlo settings
☒	☐	For hierarchical and complex designs, identification of the appropriate level for tests and full reporting of outcomes
☒	☐	Estimates of effect sizes (e.g. Cohen's *d*, Pearson's *r*), indicating how they were calculated

Our web collection on statistics for biologists contains articles on many of the points above.

Software and code

Policy information about availability of computer code

Data collection	No software for data collection was used
Data analysis	Published code<br><br>Cutadapt (version 1.15): Martin, M. et al. Cutadapt Removes Adapter Sequences From High-Throughput Sequencing Reads. EMBnet.journal 17, 10-12 (2011)<br><br>Bowtie 2 (version 2.2.9): Langmead, B. & Salzberg, SL. Fast gapped-read alignment with Bowtie 2. Nat Methods 9(4):357-9 (2012)<br><br>BWA-mem (version 0.7.16): Li, H. et al. Fast and accurate short read alignment with Burrows-Wheeler transform. Bioinformatics 25 (14):1754-60 (2009)<br><br>SAMtools (version 1.8): Li, H. et al. The Sequence Alignment/Map format and SAMtools. Bioinformatics 25(16):2078-9. (2009)<br><br>R (version 3.5.0): R Core Team. A language and environment for statistical computing. R Foundation for Statistical Computing. https:// www.R-project.org/ (2018)<br><br>R package GenomicFeatures: Lawrence, M. & Huber, W. Software for computing and annotating genomic ranges. PLoS Comput Biol 9, e1003118 (2013).<br><br>R package DESeq2 (version 1.20.0): Love, M. I., Huber, W. & Anders, S. Moderated estimation of fold change and dispersion for RNA-seq data with DESeq2. Genome Biol 15, 550 (2014)<br><br>Python (version 3.6): van Rossum, G. & de Boer, J. Interactively Testing Remote Servers Using the Python Programming Language. CWI

Quarterly 4(4):283-303. (1991)

COVERnant (version 0.3.2): https://github.com/bgbrink/COVERnant

ImageJ: https://imagej.nih.gov/ij/index.html

PEAKS studio X (Bioinformatics Solutions Inc., Canada)

GraphPad Prism version 7.0c (GraphPad Software, USA)

FIPQuant: ElBashir, R., Vanselow, J. T., Kraus, A., Janzen, C. J., Siegel, T. N., & Schlosser, A. Fragment ion patchwork quantification for measuring site-specific acetylation degrees. Anal Chem, 87(19), 9939-9945. (2015)

The RNA-seq and ChIP-seq data used in this publication have been deposited in EBI's European Nucleotide Archive and are accessible through ENA Study accession number PRJEB33216. The mass spectrometry proteomics data have been deposited to the ProteomeXchange Consortium via the PRIDE partner repository with the dataset identifier PXD014452. The Workflows and custom-made Unix Shell, Python and R scripts have been deposited at Zenodo [https://doi.org/10.5281/zenodo.3662776]. Documentation to reproduce the data analysis is provided.

For manuscripts utilizing custom algorithms or software that are central to the research but not yet described in published literature, software must be made available to editors/reviewers. We strongly encourage code deposition in a community repository (e.g. GitHub). See the Nature Research guidelines for submitting code & software for further information.

Data

Policy information about availability of data

All manuscripts must include a data availability statement. This statement should provide the following information, where applicable:
- Accession codes, unique identifiers, or web links for publicly available datasets
- A list of figures that have associated raw data
- A description of any restrictions on data availability

All sequencing data and coverage tracks generated for this publication have been deposited in the Gene Expression Omnibus (GEO) and will be accessible through the accession number GSE145812 [https://www.ncbi.nlm.nih.gov/geo/query/acc.cgi?acc=GSE145812].
Mass spectrometry proteomics data generated for this publication have been deposited in the ProteomeXchange Consortium via the PRIDE 76 partner repository and will be accessible through the accession number PXD014452 [https://www.ebi.ac.uk/pride/archive/projects/PXD014452].
The source data underlying Figures 1a–b, 2b, 3a–b, 4, 5a–b, 6a–c, 7a–c, 8a–c, and Supplementary Figures 1a–c, 10a–b, 11, 12, 13, 14a–b and 15a–b are provided as a Source Data file.

Field-specific reporting

Please select the one below that is the best fit for your research. If you are not sure, read the appropriate sections before making your selection.

☒ Life sciences ☐ Behavioural & social sciences ☐ Ecological, evolutionary & environmental sciences

For a reference copy of the document with all sections, see nature.com/documents/nr-reporting-summary-flat.pdf

Life sciences study design

All studies must disclose on these points even when the disclosure is negative.

Sample size	Sample sizes were not statistically predetermined. Whole-histone extraction and TY1-H2A immunoprecipitation experiments were performed in triplicates to rule out experimental and biological biases and to increase the resolution. TY1-H2A.Z-immunoprecipitation experiments was performed in 6 replicates, since the H2A.Z protein sequence harbors a long lysine array, which could lead to technical variations. Only one H2A.Z-immunoprecipitation experiment was performed to compare it with TY1-H2A.Z immunoprecipitation experiments. The H2A.Z immunoprecipitation experiment did not demonstrate a difference. H2A.Z-immunoprecipitation experiments after 48h HAT1 depletion were performed in triplicates to rule out experimental and biological biases and to increase the resolution. H2A.Z-immunoprecipitation experiments after 48h HAT2 depletion were performed in duplicates, since immunoprecipation of H2A.Z-containing nucleosomes did not recover complete TSS-nucleosomes.<br>RNA-seq experiments were performed in triplicates to rule out experimental biases and to be able to perform differential gene expression analysis.<br>ChIP-seq experiments were performed in duplicates to rule out experimental biases. H2A.Z-ChIP-seq experiments following 0 h and 48 h of HAT1 or HAT2 depletion were performed in quadruplicates, since they are the core experiments.<br>Experiments analyzing chromatin-bound proteins were performed in triplicates to rule out experimental biases.
Data exclusions	No data were excluded from the analyses.
Replication	All attempts to reproduce the data were successful.<br><br>Whole histone extraction experiments were performed in triplicates.<br>TY-H2A-immunoprecipitation experiments were performed in triplicates.

TY-H2A.Z-immunoprecipitation experiments was performed in 6 replicates.
Only one H2A.Z-immunoprecipitation experiment was performed demonstrating no difference to TY-H2A.Z-immunoprecipitations.
H2A.Z-immunoprecipitation experiments after 48h HAT1 depletion were performed in triplicates.
H2A.Z-immunoprecipitation experiments after 48h HAT2 depletion were performed in duplicates.
RNA-seq experiments were performed in triplicates.
Only one H2A.Z-MNase-ChIP-seq experiment in WT cells was performed reproducing previous findings.
H2A.Z-MNase-ChIP-seq experiments in HAT1-depleted cells were performed in replicates (0h: n=4; 24h: n=2; 48h: n=4) with an input control for each experiment.
H2A.Z-MNase-ChIP-seq experiments in HAT2-depleted cells were performed in replicates (0h: n=4; 24h: n=2; 48h: n=4; 72h: n=2) with an input control for each experiment, except for replicate 1.
H2A.Z-MNase-ChIP-seq experiments in HAT2-depleted cells were performed in replicates (0h: n=4; 24h: n=2; 48h: n=4; 72h: n=2) with an input control for each experiment.
TY-H2A-MNase-ChIP-seq experiments were performed in duplicates with an input control for each experiment.
RPB9-ChIP-seq experiments in HAT1 and HAT2 depleted cells were performed in duplicates with an input control for each experiment.
Experiments analyzing chromatin-bound H2A.Z after HAT2 depletion were performed in triplicates.
Experiments analyzing chromatin-bound RPB9 after HAT1 depletion were performed in triplicates.

Randomization

Not relevant for this study; Allocation of samples/organisms into experimental groups was not needed, since the experiments were performed at particular conditions.

Blinding

Not relevant for this study; Blinding was not needed, since the experiments were performed at particular conditions.

Reporting for specific materials, systems and methods

We require information from authors about some types of materials, experimental systems and methods used in many studies. Here, indicate whether each material, system or method listed is relevant to your study. If you are not sure if a list item applies to your research, read the appropriate section before selecting a response.

Materials & experimental systems

n/a	Involved in the study	
☐	☒	Antibodies
☐	☒	Eukaryotic cell lines
☒	☐	Palaeontology
☒	☐	Animals and other organisms
☒	☐	Human research participants
☒	☐	Clinical data

Methods

n/a	Involved in the study	
☐	☒	ChIP-seq
☒	☐	Flow cytometry
☒	☐	MRI-based neuroimaging

Antibodies

Antibodies used

Mouse Monoclonal BB2 antibody; purified from hybridoma cells (MAB Monoclonal Antibody Core Facility, Helmholtz Zentrum München Germany)
Bastin, P., Bagherzadeh, A., Matthews, K. R. & Gull, K. A novel epitope tag system to study protein targeting and organelle biogenesis in Trypanosoma brucei. Mol. Biochem. Parasitol. 77, 235-239 (1996).

Polyclonal Rabbit H2A.Z antibody; purified (ordered from Pineda Antikörper-Service, Berlin; available in the Siegel Lab)
Wedel, C., Förstner, K. U., Derr, R. & Siegel, T. N. GT-rich promoters can drive RNA pol II transcription and deposition of H2A.Z in African trypanosomes. EMBO J 36, 2581-2594 (2017).

Polyclonal Rabbit H3 antibody; serum (provided by Prof. C. J. Janzen; University of Würzburg)
Gassen, A., Brechtefeld, D., Schandry, N., Arteaga-Salas, J. M., Israel, L., Imhof, A. & Janzen, C. J. DOT1A-dependent H3K76 methylation is required for replication regulation in Trypanosoma brucei. Nucleic Acids 40, 10302-11 (2012).

Validation

Primary antibodies were validated regarding specificity and checked for cross-reactivity as described in the according publications:

BB2 antibody
Bastin, P., Bagherzadeh, A., Matthews, K. R. & Gull, K. A novel epitope tag system to study protein targeting and organelle biogenesis in Trypanosoma brucei. Mol. Biochem. Parasitol. 77, 235-239 (1996).

H2A.Z antibody
Wedel, C., Förstner, K. U., Derr, R. & Siegel, T. N. GT-rich promoters can drive RNA pol II transcription and deposition of H2A.Z in African trypanosomes. EMBO J 36, 2581-2594 (2017).

H3 antibody
Gassen, A., Brechtefeld, D., Schandry, N., Arteaga-Salas, J. M., Israel, L., Imhof, A. & Janzen, C. J. DOT1A-dependent H3K76

methylation is required for replication regulation in Trypanosoma brucei. Nucleic Acids 40, 10302-11 (2012).

Eukaryotic cell lines

Policy information about cell lines

Cell line source(s)	Trypanosoma brucei brucei Lister 427 was originally obtained from Prof. George Cross (Rockefeller University, NYC, USA). Subsequent genetic modifications were performed by the authors.
Authentication	RNA-seq provided authentication.
Mycoplasma contamination	Mycoplasma contamination check carried our approx. every 3 years - no positive results from those tests to date.
Commonly misidentified lines (See ICLAC register)	T. b. brucei L427 is not a commonly misidentified line.

ChIP-seq

Data deposition

☒ Confirm that both raw and final processed data have been deposited in a public database such as GEO.

☒ Confirm that you have deposited or provided access to graph files (e.g. BED files) for the called peaks.

Data access links
May remain private before publication.

Raw ChIP-seq data (.fq) used in this publication have been deposited in the Gene Expression Omnibus (GEO) and will be accessible through the accession number GSE145812 [https://www.ncbi.nlm.nih.gov/geo/query/acc.cgi?acc=GSE145812].

Partially processed datasets (.bam), workflows and custom-made Unix Shell, Python and R scripts have been deposited on our university folder and can be accessed through Zendodo [https://doi.org/10.5281/zenodo.3662776]. Documentation to reproduce the data analysis is provided.

Final processed data (coverage tracks .wig) have been deposited in the Gene Expression Omnibus (GEO) and will be accessible through the accession numberGSE145812 [https://www.ncbi.nlm.nih.gov/geo/query/acc.cgi?acc=GSE145812].

Files in database submission

H2A.Z ChIP Raw sequencing files:
NS263_WT_ChIP_H2AZ_R1.fq.bz2; NS263_WT_ChIP_H2AZ_R2.fq.bz2
NS276_2T1_HAT2_KD_ChIP_0h_1_R1.fq.gz; NS276_2T1_HAT2_KD_ChIP_0h_1_R2.fq.gz
NS277_2T1_HAT2_KD_ChIP_24h_1_R1.fq.gz; NS277_2T1_HAT2_KD_ChIP_24h_1_R2.fq.gz
NS278_2T1_HAT2_KD_ChIP_48h_1_R1.fq.gz; NS278_2T1_HAT2_KD_ChIP_48h_1_R2.fq.gz
NS283_HAT2_KD_2T1_ChIP_0h_2_R1.fq.gz; NS283_HAT2_KD_2T1_ChIP_0h_2_R2.fq.gz
NS284_HAT2_KD_2T1_ChIP_24h_2_R1.fq.gz; NS284_HAT2_KD_2T1_ChIP_24h_2_R2.fq.gz
NS285_HAT2_KD_2T1_ChIP_48h_2_R1.fq.gz; NS285_HAT2_KD_2T1_ChIP_48h_2_R2.fq.gz
NS286_HAT2_KD_2T1_ChIP_72h_1_R1.fq.gz; NS286_HAT2_KD_2T1_ChIP_72h_1_R2.fq.gz
NS287_HAT2_KD_2T1_ChIP_72h_2_R1.fq.gz; NS287_HAT2_KD_2T1_ChIP_72h_2_R2.fq.gz
NS291_HAT2_KD_2T1_Input_0h_2_R1.fq.gz; NS291_HAT2_KD_2T1_Input_0h_2_R2.fq.gz
NS292_HAT2_KD_2T1_Input_24h_2_R1.fq.gz; NS292_HAT2_KD_2T1_Input_24h_2_R2.fq.gz
NS293_HAT2_KD_2T1_Input_48h_2_R1.fq.gz; NS293_HAT2_KD_2T1_Input_48h_2_R2.fq.gz
NS294_HAT2_KD_2T1_Input_72h_1_R1.fq.gz; NS294_HAT2_KD_2T1_Input_72h_1_R2.fq.gz
NS295_HAT2_KD_2T1_Input_72h_2_R1.fq.gz; NS295_HAT2_KD_2T1_Input_72h_2_R2.fq.gz
NS476_2T1_HAT1_KD_24h_Input_1_R1.fq.gz; NS476_2T1_HAT1_KD_24h_Input_1_R2.fq.gz
NS477_2T1_HAT1_KD_48h_Input_1_R1.fq.gz; NS477_2T1_HAT1_KD_48h_Input_1_R2.fq.gz
NS484_2T1_HAT1_KD_0h_ChIP_1_R1.fq.gz; NS484_2T1_HAT1_KD_0h_ChIP_1_R2.fq.gz
NS485_2T1_HAT1_KD_24h_ChIP_1_R1.fq.gz; NS485_2T1_HAT1_KD_24h_ChIP_1_R2.fq.gz
NS486_2T1_HAT1_KD_48h_ChIP_1_R1.fq.gz; NS486_2T1_HAT1_KD_48h_ChIP_1_R2.fq.gz
NS487_2T1_HAT1_KD_0h_Input_1_R1.fq.gz; NS487_2T1_HAT1_KD_0h_Input_1_R2.fq.gz
NS491_2T1_HAT1_KD_0h_ChIP_2_R1.fq.gz; NS491_2T1_HAT1_KD_0h_ChIP_2_R2.fq.gz
NS492_2T1_HAT1_KD_24h_ChIP_2_R1.fq.gz; NS492_2T1_HAT1_KD_24h_ChIP_2_R2.fq.gz
NS493_2T1_HAT1_KD_48h_ChIP_2_R1.fq.gz; NS493_2T1_HAT1_KD_48h_ChIP_2_R2.fq.gz
NS494_2T1_HAT1_KD_0h_Input_2_R1.fq.gz; NS494_2T1_HAT1_KD_0h_Input_2_R2.fq.gz
NS495_2T1_HAT1_KD_24h_Input_2_R1.fq.gz; NS495_2T1_HAT1_KD_24h_Input_2_R2.fq.gz
NS496_2T1_HAT1_KD_48h_Input_2_R1.fq.gz; NS496_2T1_HAT1_KD_48h_Input_2_R2.fq.gz
NS531_HAT1KD_0h_H2AZ_ChIP_3_R1.fq.bz2; NS531_HAT1KD_0h_H2AZ_ChIP_3_R2.fq.bz2
NS532_HAT1KD_0h_H2AZ_ChIP_4_R1.fq.bz2; NS532_HAT1KD_0h_H2AZ_ChIP_4_R2.fq.bz2
NS533_HAT1KD_48h_H2AZ_ChIP_3_R1.fq.bz2; NS533_HAT1KD_48h_H2AZ_ChIP_3_R2.fq.bz2
NS534_HAT1KD_48h_H2AZ_ChIP_4_R1.fq.bz2; NS534_HAT1KD_48h_H2AZ_ChIP_4_R2.fq.bz2
NS535_HAT1KD_0h_H2AZ_Input_3_R1.fq.bz2; NS535_HAT1KD_0h_H2AZ_Input_3_R2.fq.bz2
NS536_HAT1KD_0h_H2AZ_Input_4_R1.fq.bz2; NS536_HAT1KD_0h_H2AZ_Input_4_R2.fq.bz2
NS537_HAT1KD_48h_H2AZ_Input_3_R1.fq.bz2; NS537_HAT1KD_48h_H2AZ_Input_3_R2.fq.bz2
NS538_HAT1KD_48h_H2AZ_Input_4_R1.fq.bz2; NS538_HAT1KD_48h_H2AZ_Input_4_R2.fq.bz2

NS539_HAT2KD_0h_H2AZ_ChIP_3_R1.fq.bz2; NS539_HAT2KD_0h_H2AZ_ChIP_3_R2.fq.bz2
NS540_HAT2KD_0h_H2AZ_ChIP_4_R1.fq.bz2; NS540_HAT2KD_0h_H2AZ_ChIP_4_R2.fq.bz2
NS541_HAT2KD_48h_H2AZ_ChIP_3_R1.fq.bz2; NS541_HAT2KD_48h_H2AZ_ChIP_3_R2.fq.bz2
NS542_HAT2KD_48h_H2AZ_ChIP_4_R1.fq.bz2; NS542_HAT2KD_48h_H2AZ_ChIP_4_R2.fq.bz2
NS543_HAT2KD_0h_H2AZ_Input_3_R1.fq.bz2; NS543_HAT2KD_0h_H2AZ_Input_3_R2.fq.bz2
NS544_HAT2KD_0h_H2AZ_Input_4_R1.fq.bz2; NS544_HAT2KD_0h_H2AZ_Input_4_R2.fq.bz2
NS545_HAT2KD_48h_H2AZ_Input_3_R1.fq.bz2; NS545_HAT2KD_48h_H2AZ_Input_3_R2.fq.bz2
NS546_HAT2KD_48h_H2AZ_Input_4_R1.fq.bz2; NS546_HAT2KD_48h_H2AZ_Input_4_R2.fq.bz2

H2A.Z ChIP processed files:
NS263_WT_ChIP_H2AZ_to_HGAP3_Tb427v9_bwt2.sorted.bam
NS276_2T1_HAT2_KD_ChIP_0h_1_to_HGAP3_Tb427v9_bwt2.sorted.bam
NS277_2T1_HAT2_KD_ChIP_24h_1_to_HGAP3_Tb427v9_bwt2.sorted.bam
NS278_2T1_HAT2_KD_ChIP_48h_1_to_HGAP3_Tb427v9_bwt2.sorted.bam
NS283_HAT2_KD_2T1_ChIP_0h_2_to_HGAP3_Tb427v9_bwt2.sorted.bam
NS284_HAT2_KD_2T1_ChIP_24h_2_to_HGAP3_Tb427v9_bwt2.sorted.bam
NS285_HAT2_KD_2T1_ChIP_48h_2_to_HGAP3_Tb427v9_bwt2.sorted.bam
NS286_HAT2_KD_2T1_ChIP_72h_1_to_HGAP3_Tb427v9_bwt2.sorted.bam
NS287_HAT2_KD_2T1_ChIP_72h_2_to_HGAP3_Tb427v9_bwt2.sorted.bam
NS291_HAT2_KD_2T1_Input_0h_2_to_HGAP3_Tb427v9_bwt2.sorted.bam
NS292_HAT2_KD_2T1_Input_24h_2_to_HGAP3_Tb427v9_bwt2.sorted.bam
NS293_HAT2_KD_2T1_Input_48h_2_to_HGAP3_Tb427v9_bwt2.sorted.bam
NS294_HAT2_KD_2T1_Input_72h_1_to_HGAP3_Tb427v9_bwt2.sorted.bam
NS295_HAT2_KD_2T1_Input_72h_2_to_HGAP3_Tb427v9_bwt2.sorted.bam
NS476_2T1_HAT1_KD_24h_Input_1_to_HGAP3_Tb427v9_bwt2.sorted.bam
NS477_2T1_HAT1_KD_48h_Input_1_to_HGAP3_Tb427v9_bwt2.sorted.bam
NS484_2T1_HAT1_KD_0h_ChIP_1_to_HGAP3_Tb427v9_bwt2.sorted.bam
NS485_2T1_HAT1_KD_24h_ChIP_1_to_HGAP3_Tb427v9_bwt2.sorted.bam
NS486_2T1_HAT1_KD_48h_ChIP_1_to_HGAP3_Tb427v9_bwt2.sorted.bam
NS487_2T1_HAT1_KD_0h_Input_1_to_HGAP3_Tb427v9_bwt2.sorted.bam
NS491_2T1_HAT1_KD_0h_ChIP_2_to_HGAP3_Tb427v9_bwt2.sorted.bam
NS492_2T1_HAT1_KD_24h_ChIP_2_to_HGAP3_Tb427v9_bwt2.sorted.bam
NS493_2T1_HAT1_KD_48h_ChIP_2_to_HGAP3_Tb427v9_bwt2.sorted.bam
NS494_2T1_HAT1_KD_0h_Input_2_to_HGAP3_Tb427v9_bwt2.sorted.bam
NS495_2T1_HAT1_KD_24h_Input_2_to_HGAP3_Tb427v9_bwt2.sorted.bam
NS496_2T1_HAT1_KD_48h_Input_2_to_HGAP3_Tb427v9_bwt2.sorted.bam
NS531_HAT1KD_0h_H2AZ_ChIP_3_to_HGAP3_Tb427v9_bwt2.sorted.bam
NS532_HAT1KD_0h_H2AZ_ChIP_4_to_HGAP3_Tb427v9_bwt2.sorted.bam
NS533_HAT1KD_48h_H2AZ_ChIP_3_to_HGAP3_Tb427v9_bwt2.sorted.bam
NS534_HAT1KD_48h_H2AZ_ChIP_4_to_HGAP3_Tb427v9_bwt2.sorted.bam
NS535_HAT1KD_0h_H2AZ_Input_3_to_HGAP3_Tb427v9_bwt2.sorted.bam
NS536_HAT1KD_0h_H2AZ_Input_4_to_HGAP3_Tb427v9_bwt2.sorted.bam
NS537_HAT1KD_48h_H2AZ_Input_3_to_HGAP3_Tb427v9_bwt2.sorted.bam
NS538_HAT1KD_48h_H2AZ_Input_4_to_HGAP3_Tb427v9_bwt2.sorted.bam
NS539_HAT2KD_0h_H2AZ_ChIP_3_to_HGAP3_Tb427v9_bwt2.sorted.bam
NS540_HAT2KD_0h_H2AZ_ChIP_4_to_HGAP3_Tb427v9_bwt2.sorted.bam
NS541_HAT2KD_48h_H2AZ_ChIP_3_to_HGAP3_Tb427v9_bwt2.sorted.bam
NS542_HAT2KD_48h_H2AZ_ChIP_4_to_HGAP3_Tb427v9_bwt2.sorted.bam
NS543_HAT2KD_0h_H2AZ_Input_3_to_HGAP3_Tb427v9_bwt2.sorted.bam
NS544_HAT2KD_0h_H2AZ_Input_4_to_HGAP3_Tb427v9_bwt2.sorted.bam
NS545_HAT2KD_48h_H2AZ_Input_3_to_HGAP3_Tb427v9_bwt2.sorted.bam
NS546_HAT2KD_48h_H2AZ_Input_4_to_HGAP3_Tb427v9_bwt2.sorted.bam

H2A.Z ChIP coverage:
CpB_ws5001_ss1001_ratio_NS565_SM_TyH2A_Input_1_to_HGAP3_Tb427v9_bwt2.sorted_vs_NS263_WT_ChIP_H2AZ_to_HGAP3_Tb427v9_bwt2.sorted.wig;
CpB_ws5001_ss1001_numerator_NS263_WT_ChIP_H2AZ_to_HGAP3_Tb427v9_bwt2.sorted.wig
CpB_ws5001_ss1001_numerator_NS285_HAT2_KD_2T1_ChIP_48h_2_to_HGAP3_Tb427v9_bwt2.sorted.wig
CpB_ws5001_ss1001_numerator_NS486_2T1_HAT1_KD_48h_ChIP_1_to_HGAP3_Tb427v9_bwt2.sorted.wig
CpB_ws5001_ss1001_ratio_NS565_SM_TyH2A_Input_1_to_HGAP3_Tb427v9_bwt2.sorted_vs_NS563_SM_TyH2A_ChIP_1_to_HGAP3_Tb427v9_bwt2.sorted.wig
CpB_ws5001_ss1001_ratio_NS565_SM_TyH2A_Input_1_to_HGAP3_Tb427v9_bwt2.sorted_vs_NS263_WT_ChIP_H2AZ_to_HGAP3_Tb427v9_bwt2.sorted.wig;
CpB_ws5001_ss1001_ratio_NS565_SM_TyH2A_Input_1_to_HGAP3_Tb427v9_bwt2.sorted_vs_NS563_SM_TyH2A_ChIP_1_to_HGAP3_Tb427v9_bwt2.sorted.wig

CpB_ws1_ss1_numerator_NS263_WT_ChIP_H2AZ_to_HGAP3_Tb427v9_bwt2.sorted.wig
CpB_ws1_ss1_numerator_NS276_2T1_HAT2_KD_ChIP_0h_1_to_HGAP3_Tb427v9_bwt2.sorted.wig

CpB_ws1_ss1_numerator_NS277_2T1_HAT2_KD_ChIP_24h_1_to_HGAP3_Tb427v9_bwt2.sorted.wig
CpB_ws1_ss1_numerator_NS278_2T1_HAT2_KD_ChIP_48h_1_to_HGAP3_Tb427v9_bwt2.sorted.wig
CpB_ws1_ss1_numerator_NS283_HAT2_KD_2T1_ChIP_0h_2_to_HGAP3_Tb427v9_bwt2.sorted.wig
CpB_ws1_ss1_numerator_NS284_HAT2_KD_2T1_ChIP_24h_2_to_HGAP3_Tb427v9_bwt2.sorted.wig
CpB_ws1_ss1_numerator_NS285_HAT2_KD_2T1_ChIP_48h_2_to_HGAP3_Tb427v9_bwt2.sorted.wig
CpB_ws1_ss1_numerator_NS286_HAT2_KD_2T1_ChIP_72h_1_to_HGAP3_Tb427v9_bwt2.sorted.wig
CpB_ws1_ss1_numerator_NS287_HAT2_KD_2T1_ChIP_72h_2_to_HGAP3_Tb427v9_bwt2.sorted.wig
CpB_ws1_ss1_denominator_NS291_HAT2_KD_2T1_Input_0h_2_to_HGAP3_Tb427v9_bwt2.sorted.wig
CpB_ws1_ss1_denominator_NS292_HAT2_KD_2T1_Input_24h_2_to_HGAP3_Tb427v9_bwt2.sorted.wig
CpB_ws1_ss1_denominator_NS293_HAT2_KD_2T1_Input_48h_2_to_HGAP3_Tb427v9_bwt2.sorted.wig
CpB_ws1_ss1_denominator_NS294_HAT2_KD_2T1_Input_72h_1_to_HGAP3_Tb427v9_bwt2.sorted.wig
CpB_ws1_ss1_denominator_NS295_HAT2_KD_2T1_Input_72h_2_to_HGAP3_Tb427v9_bwt2.sorted.wig
CpB_ws1_ss1_denominator_NS476_2T1_HAT1_KD_24h_Input_1_to_HGAP3_Tb427v9_bwt2.sorted.wig
CpB_ws1_ss1_denominator_NS477_2T1_HAT1_KD_48h_Input_1_to_HGAP3_Tb427v9_bwt2.sorted.wig
CpB_ws1_ss1_numerator_NS484_2T1_HAT1_KD_0h_ChIP_1_to_HGAP3_Tb427v9_bwt2.sorted.wig
CpB_ws1_ss1_numerator_NS485_2T1_HAT1_KD_24h_ChIP_1_to_HGAP3_Tb427v9_bwt2.sorted.wig
CpB_ws1_ss1_numerator_NS486_2T1_HAT1_KD_48h_ChIP_1_to_HGAP3_Tb427v9_bwt2.sorted.wig
CpB_ws1_ss1_denominator_NS487_2T1_HAT1_KD_0h_Input_1_to_HGAP3_Tb427v9_bwt2.sorted.wig
CpB_ws1_ss1_numerator_NS491_2T1_HAT1_KD_0h_ChIP_2_to_HGAP3_Tb427v9_bwt2.sorted.wig
CpB_ws1_ss1_numerator_NS492_2T1_HAT1_KD_24h_ChIP_2_to_HGAP3_Tb427v9_bwt2.sorted.wig
CpB_ws1_ss1_numerator_NS493_2T1_HAT1_KD_48h_ChIP_2_to_HGAP3_Tb427v9_bwt2.sorted.wig
CpB_ws1_ss1_denominator_NS494_2T1_HAT1_KD_0h_Input_2_to_HGAP3_Tb427v9_bwt2.sorted.wig
CpB_ws1_ss1_denominator_NS495_2T1_HAT1_KD_24h_Input_2_to_HGAP3_Tb427v9_bwt2.sorted.wig
CpB_ws1_ss1_denominator_NS496_2T1_HAT1_KD_48h_Input_2_to_HGAP3_Tb427v9_bwt2.sorted.wig
CpB_ws1_ss1_numerator_NS531_HAT1KD_0h_H2AZ_ChIP_3_to_HGAP3_Tb427v9_bwt2.sorted.wig
CpB_ws1_ss1_numerator_NS532_HAT1KD_0h_H2AZ_ChIP_4_to_HGAP3_Tb427v9_bwt2.sorted.wig
CpB_ws1_ss1_numerator_NS533_HAT1KD_48h_H2AZ_ChIP_3_to_HGAP3_Tb427v9_bwt2.sorted.wig
CpB_ws1_ss1_numerator_NS534_HAT1KD_48h_H2AZ_ChIP_4_to_HGAP3_Tb427v9_bwt2.sorted.wig
CpB_ws1_ss1_denominator_NS535_HAT1KD_0h_H2AZ_Input_3_to_HGAP3_Tb427v9_bwt2.sorted.wig
CpB_ws1_ss1_denominator_NS536_HAT1KD_0h_H2AZ_Input_4_to_HGAP3_Tb427v9_bwt2.sorted.wig
CpB_ws1_ss1_denominator_NS537_HAT1KD_48h_H2AZ_Input_3_to_HGAP3_Tb427v9_bwt2.sorted.wig
CpB_ws1_ss1_denominator_NS538_HAT1KD_48h_H2AZ_Input_4_to_HGAP3_Tb427v9_bwt2.sorted.wig
CpB_ws1_ss1_numerator_NS539_HAT2KD_0h_H2AZ_ChIP_3_to_HGAP3_Tb427v9_bwt2.sorted.wig
CpB_ws1_ss1_numerator_NS540_HAT2KD_0h_H2AZ_ChIP_4_to_HGAP3_Tb427v9_bwt2.sorted.wig
CpB_ws1_ss1_numerator_NS541_HAT2KD_48h_H2AZ_ChIP_3_to_HGAP3_Tb427v9_bwt2.sorted.wig
CpB_ws1_ss1_numerator_NS542_HAT2KD_48h_H2AZ_ChIP_4_to_HGAP3_Tb427v9_bwt2.sorted.wig
CpB_ws1_ss1_denominator_NS543_HAT2KD_0h_H2AZ_Input_3_to_HGAP3_Tb427v9_bwt2.sorted.wig
CpB_ws1_ss1_denominator_NS544_HAT2KD_0h_H2AZ_Input_4_to_HGAP3_Tb427v9_bwt2.sorted.wig
CpB_ws1_ss1_denominator_NS545_HAT2KD_48h_H2AZ_Input_3_to_HGAP3_Tb427v9_bwt2.sorted.wig
CpB_ws1_ss1_denominator_NS546_HAT2KD_48h_H2AZ_Input_4_to_HGAP3_Tb427v9_bwt2.sorted.wig

RNAPII ChIP raw sequencing files:
NS470_BF_2T1_TyRPB9_ChIP_1_R1.fq.gz; NS470_BF_2T1_TyRPB9_ChIP_1_R2.fq.gz
NS471_BF_2T1_TyRPB9_Input_1_R1.fq.gz; NS471_BF_2T1_TyRPB9_Input_1_R2.fq.gz
NS474_BF_2T1_TyRPB9_ChIP_2_R1.fq.gz; NS474_BF_2T1_TyRPB9_ChIP_2_R2.fq.gz
NS475_BF_2T1_TyRPB9_Input_2_R1.fq.gz; NS475_BF_2T1_TyRPB9_Input_2_R2.fq.gz
NS512_HAT1_KD_TyRPB9_0h_Input_1_R1.fq.bz2; NS512_HAT1_KD_TyRPB9_0h_Input_1_R2.fq.bz2
NS513_HAT1_KD_TyRPB9_24h_Input_1_R1.fq.bz2; NS513_HAT1_KD_TyRPB9_24h_Input_1_R2.fq.bz2
NS514_HAT1_KD_TyRPB9_48h_Input_1_R1.fq.bz2; NS514_HAT1_KD_TyRPB9_48h_Input_1_R2.fq.bz2
NS515_HAT2_KD_TyRPB9_0h_Input_1_R1.fq.bz2; NS515_HAT2_KD_TyRPB9_0h_Input_1_R2.fq.bz2
NS516_HAT2_KD_TyRPB9_24h_Input_1_R1.fq.bz2; NS516_HAT2_KD_TyRPB9_24h_Input_1_R2.fq.bz2
NS517_HAT2_KD_TyRPB9_48h_Input_1_R1.fq.bz2; NS517_HAT2_KD_TyRPB9_48h_Input_1_R2.fq.bz2
NS518_HAT1_KD_TyRPB9_0h_ChIP_1_R1.fq.bz2; NS518_HAT1_KD_TyRPB9_0h_ChIP_1_R2.fq.bz2
NS519_HAT1_KD_TyRPB9_24h_ChIP_1_R1.fq.bz2; NS519_HAT1_KD_TyRPB9_24h_ChIP_1_R2.fq.bz2
NS520_HAT1_KD_TyRPB9_48h_ChIP_1_R1.fq.bz2; NS520_HAT1_KD_TyRPB9_48h_ChIP_1_R2.fq.bz2
NS521_HAT2_KD_TyRPB9_0h_ChIP_1_R1.fq.bz2; NS521_HAT2_KD_TyRPB9_0h_ChIP_1_R2.fq.bz2
NS522_HAT2_KD_TyRPB9_24h_ChIP_1_R1.fq.bz2; NS522_HAT2_KD_TyRPB9_24h_ChIP_1_R2.fq.bz2
NS523_HAT2_KD_TyRPB9_48h_ChIP_1_R1.fq.bz2; NS523_HAT2_KD_TyRPB9_48h_ChIP_1_R2.fq.bz2
NS567_2T1_TyRPB9_HAT1KD_0h_ChIP_2_R1.fq.bz2; NS567_2T1_TyRPB9_HAT1KD_0h_ChIP_2_R2.fq.bz2
NS568_2T1_TyRPB9_HAT1KD_24h_ChIP_2_R1.fq.bz2; NS568_2T1_TyRPB9_HAT1KD_24h_ChIP_2_R2.fq.bz2
NS569_2T1_TyRPB9_HAT1KD_48h_ChIP_2_R1.fq.bz2; NS569_2T1_TyRPB9_HAT1KD_48h_ChIP_2_R2.fq.bz2
NS570_2T1_TyRPB9_HAT1KD_0h_Input_2_R1.fq.bz2; NS570_2T1_TyRPB9_HAT1KD_0h_Input_2_R2.fq.bz2
NS571_2T1_TyRPB9_HAT1KD_24h_Input_2_R1.fq.bz2; NS571_2T1_TyRPB9_HAT1KD_24h_Input_2_R2.fq.bz2
NS572_2T1_TyRPB9_HAT1KD_48h_Input_2_R1.fq.bz2; NS572_2T1_TyRPB9_HAT1KD_48h_Input_2_R2.fq.bz2
NS573_2T1_TyRPB9_HAT2KD_0h_ChIP_2_R1.fq.bz2; NS573_2T1_TyRPB9_HAT2KD_0h_ChIP_2_R2.fq.bz2
NS574_2T1_TyRPB9_HAT2KD_24h_ChIP_2_R1.fq.bz2; NS574_2T1_TyRPB9_HAT2KD_24h_ChIP_2_R2.fq.bz2
NS575_2T1_TyRPB9_HAT2KD_48h_ChIP_2_R1.fq.bz2; NS575_2T1_TyRPB9_HAT2KD_48h_ChIP_2_R2.fq.bz2
NS578_2T1_TyRPB9_HAT2KD_0h_Input_2_R1.fq.bz2; NS578_2T1_TyRPB9_HAT2KD_0h_Input_2_R2.fq.bz2

NS579_2T1_TyRPB9_HAT2KD_24h_Input_2_R1.fq.bz2; NS579_2T1_TyRPB9_HAT2KD_24h_Input_2_R2.fq.bz2
NS580_2T1_TyRPB9_HAT2KD_48h_Input_2_R1.fq.bz2; NS580_2T1_TyRPB9_HAT2KD_48h_Input_2_R2.fq.bz2

RNA Pol II ChIP processed files:
NS470_BF_2T1_TyRPB9_ChIP_1_to_HGAP3_Tb427v9_bwt2.sorted.bam
NS471_BF_2T1_TyRPB9_Input_1_to_HGAP3_Tb427v9_bwt2.sorted.bam
NS474_BF_2T1_TyRPB9_ChIP_2_to_HGAP3_Tb427v9_bwt2.sorted.bam
NS475_BF_2T1_TyRPB9_Input_2_to_HGAP3_Tb427v9_bwt2.sorted.bam
NS512_HAT1_KD_TyRPB9_0h_Input_1_to_HGAP3_Tb427v9_bwt2.sorted.bam
NS513_HAT1_KD_TyRPB9_24h_Input_1_to_HGAP3_Tb427v9_bwt2.sorted.bam
NS514_HAT1_KD_TyRPB9_48h_Input_1_to_HGAP3_Tb427v9_bwt2.sorted.bam
NS515_HAT2_KD_TyRPB9_0h_Input_1_to_HGAP3_Tb427v9_bwt2.sorted.bam
NS516_HAT2_KD_TyRPB9_24h_Input_1_to_HGAP3_Tb427v9_bwt2.sorted.bam
NS517_HAT2_KD_TyRPB9_48h_Input_1_to_HGAP3_Tb427v9_bwt2.sorted.bam
NS518_HAT1_KD_TyRPB9_0h_ChIP_1_to_HGAP3_Tb427v9_bwt2.sorted.bam
NS519_HAT1_KD_TyRPB9_24h_ChIP_1_to_HGAP3_Tb427v9_bwt2.sorted.bam
NS520_HAT1_KD_TyRPB9_48h_ChIP_1_to_HGAP3_Tb427v9_bwt2.sorted.bam
NS521_HAT2_KD_TyRPB9_0h_ChIP_1_to_HGAP3_Tb427v9_bwt2.sorted.bam
NS522_HAT2_KD_TyRPB9_24h_ChIP_1_to_HGAP3_Tb427v9_bwt2.sorted.bam
NS523_HAT2_KD_TyRPB9_48h_ChIP_1_to_HGAP3_Tb427v9_bwt2.sorted.bam
NS567_2T1_TyRPB9_HAT1KD_0h_ChIP_2_to_HGAP3_Tb427v9_bwt2.sorted.bam
NS568_2T1_TyRPB9_HAT1KD_24h_ChIP_2_to_HGAP3_Tb427v9_bwt2.sorted.bam
NS569_2T1_TyRPB9_HAT1KD_48h_ChIP_2_to_HGAP3_Tb427v9_bwt2.sorted.bam
NS570_2T1_TyRPB9_HAT1KD_0h_Input_2_to_HGAP3_Tb427v9_bwt2.sorted.bam
NS571_2T1_TyRPB9_HAT1KD_24h_Input_2_to_HGAP3_Tb427v9_bwt2.sorted.bam
NS572_2T1_TyRPB9_HAT1KD_48h_Input_2_to_HGAP3_Tb427v9_bwt2.sorted.bam
NS573_2T1_TyRPB9_HAT2KD_0h_ChIP_2_to_HGAP3_Tb427v9_bwt2.sorted.bam
NS574_2T1_TyRPB9_HAT2KD_24h_ChIP_2_to_HGAP3_Tb427v9_bwt2.sorted.bam
NS575_2T1_TyRPB9_HAT2KD_48h_ChIP_2_to_HGAP3_Tb427v9_bwt2.sorted.bam
NS578_2T1_TyRPB9_HAT2KD_0h_Input_2_to_HGAP3_Tb427v9_bwt2.sorted.bam
NS579_2T1_TyRPB9_HAT2KD_24h_Input_2_to_HGAP3_Tb427v9_bwt2.sorted.bam
NS580_2T1_TyRPB9_HAT2KD_48h_Input_2_to_HGAP3_Tb427v9_bwt2.sorted.bam

RNA Pol II ChIP coverage files:
CpB_ws1_ss1_ratio_NS471_BF_2T1_TyRPB9_Input_1_to_HGAP3_Tb427v9_bwt2.sorted_vs_NS470_BF_2T1_TyRPB9_ChIP_1_to_HGAP3_Tb427v9_bwt2.sorted.wig
CpB_ws1_ss1_ratio_NS475_BF_2T1_TyRPB9_Input_2_to_HGAP3_Tb427v9_bwt2.sorted_vs_NS474_BF_2T1_TyRPB9_ChIP_2_to_HGAP3_Tb427v9_bwt2.sorted.wig
CpB_ws1_ss1_ratio_NS512_HAT1_KD_TyRPB9_0h_Input_1_to_HGAP3_Tb427v9_bwt2.sorted_vs_NS518_HAT1_KD_TyRPB9_0h_ChIP_1_to_HGAP3_Tb427v9_bwt2.sorted.wig
CpB_ws1_ss1_ratio_NS513_HAT1_KD_TyRPB9_24h_Input_1_to_HGAP3_Tb427v9_bwt2.sorted_vs_NS519_HAT1_KD_TyRPB9_24h_ChIP_1_to_HGAP3_Tb427v9_bwt2.sorted.wig
CpB_ws1_ss1_ratio_NS514_HAT1_KD_TyRPB9_48h_Input_1_to_HGAP3_Tb427v9_bwt2.sorted_vs_NS520_HAT1_KD_TyRPB9_48h_ChIP_1_to_HGAP3_Tb427v9_bwt2.sorted.wig
CpB_ws1_ss1_ratio_NS515_HAT2_KD_TyRPB9_0h_Input_1_to_HGAP3_Tb427v9_bwt2.sorted_vs_NS521_HAT2_KD_TyRPB9_0h_ChIP_1_to_HGAP3_Tb427v9_bwt2.sorted.wig
CpB_ws1_ss1_ratio_NS516_HAT2_KD_TyRPB9_24h_Input_1_to_HGAP3_Tb427v9_bwt2.sorted_vs_NS522_HAT2_KD_TyRPB9_24h_ChIP_1_to_HGAP3_Tb427v9_bwt2.sorted.wig
CpB_ws1_ss1_ratio_NS517_HAT2_KD_TyRPB9_48h_Input_1_to_HGAP3_Tb427v9_bwt2.sorted_vs_NS523_HAT2_KD_TyRPB9_48h_ChIP_1_to_HGAP3_Tb427v9_bwt2.sorted.wig
CpB_ws1_ss1_ratio_NS512_HAT1_KD_TyRPB9_0h_Input_1_to_HGAP3_Tb427v9_bwt2.sorted_vs_NS518_HAT1_KD_TyRPB9_0h_ChIP_1_to_HGAP3_Tb427v9_bwt2.sorted.wig
CpB_ws1_ss1_ratio_NS513_HAT1_KD_TyRPB9_24h_Input_1_to_HGAP3_Tb427v9_bwt2.sorted_vs_NS519_HAT1_KD_TyRPB9_24h_ChIP_1_to_HGAP3_Tb427v9_bwt2.sorted.wig
CpB_ws1_ss1_ratio_NS514_HAT1_KD_TyRPB9_48h_Input_1_to_HGAP3_Tb427v9_bwt2.sorted_vs_NS520_HAT1_KD_TyRPB9_48h_ChIP_1_to_HGAP3_Tb427v9_bwt2.sorted.wig
CpB_ws1_ss1_ratio_NS515_HAT2_KD_TyRPB9_0h_Input_1_to_HGAP3_Tb427v9_bwt2.sorted_vs_NS521_HAT2_KD_TyRPB9_0h_ChIP_1_to_HGAP3_Tb427v9_bwt2.sorted.wig
CpB_ws1_ss1_ratio_NS516_HAT2_KD_TyRPB9_24h_Input_1_to_HGAP3_Tb427v9_bwt2.sorted_vs_NS522_HAT2_KD_TyRPB9_24h_ChIP_1_to_HGAP3_Tb427v9_bwt2.sorted.wig
CpB_ws1_ss1_ratio_NS517_HAT2_KD_TyRPB9_48h_Input_1_to_HGAP3_Tb427v9_bwt2.sorted_vs_NS523_HAT2_KD_TyRPB9_48h_ChIP_1_to_HGAP3_Tb427v9_bwt2.sorted.wig
CpB_ws1_ss1_ratio_NS570_2T1_TyRPB9_HAT1KD_0h_Input_2_to_HGAP3_Tb427v9_bwt2.sorted_vs_NS567_2T1_TyRPB9_HAT1KD_0h_ChIP_2_to_HGAP3_Tb427v9_bwt2.sorted.wig
CpB_ws1_ss1_ratio_NS571_2T1_TyRPB9_HAT1KD_24h_Input_2_to_HGAP3_Tb427v9_bwt2.sorted_vs_NS568_2T1_TyRPB9_HAT1KD_24h_ChIP_2_to_HGAP3_Tb427v9_bwt2.sorted.wig
CpB_ws1_ss1_ratio_NS572_2T1_TyRPB9_HAT1KD_48h_Input_2_to_HGAP3_Tb427v9_bwt2.sorted_vs_NS569_2T1_TyRPB9_HAT1KD_48h_ChIP_2_to_HGAP3_Tb427v9_bwt2.sorted.wig
CpB_ws1_ss1_ratio_NS570_2T1_TyRPB9_HAT1KD_0h_Input_2_to_HGAP3_Tb427v9_bwt2.sorted_vs_NS567_2T1_TyRPB9_

HAT1KD_0h_ChIP_2_to_HGAP3_Tb427v9_bwt2.sorted.wig
CpB_ws1_ss1_ratio_NS571_2T1_TyRPB9_HAT1KD_24h_Input_2_to_HGAP3_Tb427v9_bwt2.sorted_vs_NS568_2T1_TyRPB9_HAT1KD_24h_ChIP_2_to_HGAP3_Tb427v9_bwt2.sorted.wig
CpB_ws1_ss1_ratio_NS572_2T1_TyRPB9_HAT1KD_48h_Input_2_to_HGAP3_Tb427v9_bwt2.sorted_vs_NS569_2T1_TyRPB9_HAT1KD_48h_ChIP_2_to_HGAP3_Tb427v9_bwt2.sorted.wig
CpB_ws1_ss1_ratio_NS578_2T1_TyRPB9_HAT2KD_0h_Input_2_to_HGAP3_Tb427v9_bwt2.sorted_vs_NS573_2T1_TyRPB9_HAT2KD_0h_ChIP_2_to_HGAP3_Tb427v9_bwt2.sorted.wig
CpB_ws1_ss1_ratio_NS579_2T1_TyRPB9_HAT2KD_24h_Input_2_to_HGAP3_Tb427v9_bwt2.sorted_vs_NS574_2T1_TyRPB9_HAT2KD_24h_ChIP_2_to_HGAP3_Tb427v9_bwt2.sorted.wig
CpB_ws1_ss1_ratio_NS580_2T1_TyRPB9_HAT2KD_48h_Input_2_to_HGAP3_Tb427v9_bwt2.sorted_vs_NS575_2T1_TyRPB9_HAT2KD_48h_ChIP_2_to_HGAP3_Tb427v9_bwt2.sorted.wig
CpB_ws1_ss1_ratio_NS578_2T1_TyRPB9_HAT2KD_0h_Input_2_to_HGAP3_Tb427v9_bwt2.sorted_vs_NS573_2T1_TyRPB9_HAT2KD_0h_ChIP_2_to_HGAP3_Tb427v9_bwt2.sorted.wig
CpB_ws1_ss1_ratio_NS579_2T1_TyRPB9_HAT2KD_24h_Input_2_to_HGAP3_Tb427v9_bwt2.sorted_vs_NS574_2T1_TyRPB9_HAT2KD_24h_ChIP_2_to_HGAP3_Tb427v9_bwt2.sorted.wig
CpB_ws1_ss1_ratio_NS580_2T1_TyRPB9_HAT2KD_48h_Input_2_to_HGAP3_Tb427v9_bwt2.sorted_vs_NS575_2T1_TyRPB9_HAT2KD_48h_ChIP_2_to_HGAP3_Tb427v9_bwt2.sorted.wig

Genome browser session
(e.g. UCSC)

Not used

Methodology

Replicates

Only one H2A.Z-MNase-ChIP-seq experiment in WT cells was performed to reproduce previous findings.
H2A.Z-MNase-ChIP-seq experiments in HAT1-depleted cells were performed in replicates (0h: n=4; 24h: n=2; 48h: n=4) with an input control for each experiment.
H2A.Z-MNase-ChIP-seq experiments in HAT2-depleted cells were performed in replicates (0h: n=4; 24h: n=2; 48h: n=4; 72h: n=2) with an input control for each experiment, except for replicate 1.
H2A.Z-MNase-ChIP-seq experiments in HAT2-depleted cells were performed in replicates (0h: n=4; 24h: n=2; 48h: n=4; 72h: n=2) with an input control for each experiment.
TY-H2A-MNase-ChIP-seq experiments were performed in duplicates with an input control for each experiment.
RPB9-ChIP-seq experiments in HAT1 and HAT2 depleted cells were performed in duplicates with an input control for each experiment.

Sequencing depth

"NS263_WT_ChIP_H2AZ_R1.fq.bz2; NS263_WT_ChIP_H2AZ_R2.fq.bz2 ";Illumina NextSeq 500, paired-end, 2x76 bp;sequenced reads: 3015378;Bowtie2 version 2.2.9, local;mapping rate: 99.08%
"NS276_2T1_HAT2_KD_ChIP_0h_1_R1.fq.gz; NS276_2T1_HAT2_KD_ChIP_0h_1_R2.fq.gz";Illumina NextSeq 500, paired-end, 2x76 bp;sequenced reads: 3380265;Bowtie2 version 2.2.9, local;mapping rate: 97.95%
"NS277_2T1_HAT2_KD_ChIP_24h_1_R1.fq.gz; NS277_2T1_HAT2_KD_ChIP_24h_1_R2.fq.gz";Illumina NextSeq 500, paired-end, 2x76 bp;sequenced reads: 3357305;Bowtie2 version 2.2.9, local;mapping rate: 98.39%
"NS278_2T1_HAT2_KD_ChIP_48h_1_R1.fq.gz; NS278_2T1_HAT2_KD_ChIP_48h_1_R2.fq.gz";Illumina NextSeq 500, paired-end, 2x76 bp;sequenced reads: 3453220;Bowtie2 version 2.2.9, local;mapping rate: 98.62%
"NS283_HAT2_KD_2T1_ChIP_0h_2_R1.fq.gz; NS283_HAT2_KD_2T1_ChIP_0h_2_R2.fq.gz";Illumina NextSeq 500, paired-end, 2x76 bp;sequenced reads: 2619228;Bowtie2 version 2.2.9, local;mapping rate: 97.67%
"NS284_HAT2_KD_2T1_ChIP_24h_2_R1.fq.gz; NS284_HAT2_KD_2T1_ChIP_24h_2_R2.fq.gz";Illumina NextSeq 500, paired-end, 2x76 bp;sequenced reads: 2695096;Bowtie2 version 2.2.9, local;mapping rate: 96.62%
"NS285_HAT2_KD_2T1_ChIP_48h_2_R1.fq.gz; NS285_HAT2_KD_2T1_ChIP_48h_2_R2.fq.gz";Illumina NextSeq 500, paired-end, 2x76 bp;sequenced reads: 2276000;Bowtie2 version 2.2.9, local; mapping rate: 95.92%
"NS286_HAT2_KD_2T1_ChIP_72h_1_R1.fq.gz; NS286_HAT2_KD_2T1_ChIP_72h_1_R2.fq.gz";Illumina NextSeq 500, paired-end, 2x76 bp;sequenced reads: 1080736;Bowtie2 version 2.2.9, local;mapping rate: 96.91%
"NS287_HAT2_KD_2T1_ChIP_72h_2_R1.fq.gz; NS287_HAT2_KD_2T1_ChIP_72h_2_R2.fq.gz";Illumina NextSeq 500, paired-end, 2x76 bp; sequenced reads: 7344525;Bowtie2 version 2.2.9, local;mapping rate: 97.61%
"NS291_HAT2_KD_2T1_Input_0h_2_R1.fq.gz; NS291_HAT2_KD_2T1_Input_0h_2_R2.fq.gz ";Illumina NextSeq 500, paired-end, 2x76 bp;sequenced reads: 2788620;Bowtie2 version 2.2.9, local;mapping rate: 93.45%
"NS292_HAT2_KD_2T1_Input_24h_2_R1.fq.gz; NS292_HAT2_KD_2T1_Input_24h_2_R2.fq.gz";Illumina NextSeq 500, paired-end, 2x76 bp;sequenced reads: 2709181;Bowtie2 version 2.2.9, local;mapping rate: 96.05%
"NS293_HAT2_KD_2T1_Input_48h_2_R1.fq.gz; NS293_HAT2_KD_2T1_Input_48h_2_R2.fq.gz";Illumina NextSeq 500, paired-end, 2x76 bp;sequenced reads: 3004490;Bowtie2 version 2.2.9, local;mapping rate: 94.67%
"NS294_HAT2_KD_2T1_Input_72h_1_R1.fq.gz; NS294_HAT2_KD_2T1_Input_72h_1_R2.fq.gz";Illumina NextSeq 500, paired-end, 2x76 bp;sequenced reads: 2816922;Bowtie2 version 2.2.9, local;mapping rate: 95.57%
"NS295_HAT2_KD_2T1_Input_72h_2_R1.fq.gz; NS295_HAT2_KD_2T1_Input_72h_2_R2.fq.gz";Illumina NextSeq 500, paired-end, 2x76 bp;sequenced reads: 2084397;Bowtie2 version 2.2.9, local;mapping rate: 91.39%
"NS476_2T1_HAT1_KD_24h_Input_1_R1.fq.gz; NS476_2T1_HAT1_KD_24h_Input_1_R2.fq.gz";Illumina NextSeq 500, paired-end, 2x76 bp;sequenced reads: 7211097;Bowtie2 version 2.2.9, local;mapping rate: 97.38%
"NS477_2T1_HAT1_KD_48h_Input_1_R1.fq.gz; NS477_2T1_HAT1_KD_48h_Input_1_R2.fq.gz";Illumina NextSeq 500, paired-end, 2x76 bp;sequenced reads: 6611792;Bowtie2 version 2.2.9, local;mapping rate: 96.91%
"NS484_2T1_HAT1_KD_0h_ChIP_1_R1.fq.gz; NS484_2T1_HAT1_KD_0h_ChIP_1_R2.fq.gz";Illumina NextSeq 500, paired-end, 2x76 bp;sequenced reads: 8091270;Bowtie2 version 2.2.9, local;mapping rate: 97.65%
"NS485_2T1_HAT1_KD_24h_ChIP_1_R1.fq.gz; NS485_2T1_HAT1_KD_24h_ChIP_1_R2.fq.gz";Illumina NextSeq 500, paired-end, 2x76 bp;sequenced reads: 8246854;Bowtie2 version 2.2.9, local;mapping rate: 97.97%
"NS486_2T1_HAT1_KD_48h_ChIP_1_R1.fq.gz; NS486_2T1_HAT1_KD_48h_ChIP_1_R2.fq.gz";Illumina NextSeq 500, paired-

end, 2x76 bp;sequenced reads: 11620011;Bowtie2 version 2.2.9, local;mapping rate: 97.97%
"NS487_2T1_HAT1_KD_0h_Input_1_R1.fq.gz; NS487_2T1_HAT1_KD_0h_Input_1_R2.fq.gz";Illumina NextSeq 500, paired-end, 2x76 bp;sequenced reads: 10018772;Bowtie2 version 2.2.9, local;mapping rate: 97.73%
"NS491_2T1_HAT1_KD_0h_ChIP_2_R1.fq.gz; NS491_2T1_HAT1_KD_0h_ChIP_2_R2.fq.gz";Illumina NextSeq 500, paired-end, 2x76 bp;sequenced reads: 3980805;Bowtie2 version 2.2.9, local;mapping rate: 97.99%
"NS492_2T1_HAT1_KD_24h_ChIP_2_R1.fq.gz; NS492_2T1_HAT1_KD_24h_ChIP_2_R2.fq.gz";Illumina NextSeq 500, paired-end, 2x76 bp;sequenced reads: 4484259;Bowtie2 version 2.2.9, local;mapping rate: 99.1%
"NS493_2T1_HAT1_KD_48h_ChIP_2_R1.fq.gz; NS493_2T1_HAT1_KD_48h_ChIP_2_R2.fq.gz";Illumina NextSeq 500, paired-end, 2x76 bp;sequenced reads: 12761757;Bowtie2 version 2.2.9, local;mapping rate: 97.75%
"NS494_2T1_HAT1_KD_0h_Input_2_R1.fq.gz; NS494_2T1_HAT1_KD_0h_Input_2_R2.fq.gz";Illumina NextSeq 500, paired-end, 2x76 bp;sequenced reads: 5350241;Bowtie2 version 2.2.9, local;mapping rate: 96.52%
"NS495_2T1_HAT1_KD_24h_Input_2_R1.fq.gz; NS495_2T1_HAT1_KD_24h_Input_2_R2.fq.gz";Illumina NextSeq 500, paired-end, 2x76 bp;sequenced reads: 5922089;Bowtie2 version 2.2.9, local; mapping rate: 96.69%
"NS496_2T1_HAT1_KD_48h_Input_2_R1.fq.gz; NS496_2T1_HAT1_KD_48h_Input_2_R2.fq.gz";Illumina NextSeq 500, paired-end, 2x76 bp;sequenced reads: 5596528;Bowtie2 version 2.2.9, local;mapping rate: 97.81%
"NS531_HAT1KD_0h_H2AZ_ChIP_3_R1.fq.bz2; NS531_HAT1KD_0h_H2AZ_ChIP_3_R2.fq.bz2";Illumina NextSeq 500, paired-end, 2x76 bp;sequenced reads: 2967118;Bowtie2 version 2.2.9, local;mapping rate: 98.04%
"NS532_HAT1KD_0h_H2AZ_ChIP_4_R1.fq.bz2; NS532_HAT1KD_0h_H2AZ_ChIP_4_R2.fq.bz2";Illumina NextSeq 500, paired-end, 2x76 bp;sequenced reads: 3597753;Bowtie2 version 2.2.9, local;mapping rate: 98.24%
"NS533_HAT1KD_48h_H2AZ_ChIP_3_R1.fq.bz2; NS533_HAT1KD_48h_H2AZ_ChIP_3_R2.fq.bz2";Illumina NextSeq 500, paired-end, 2x76 bp;sequenced reads: 3738898;Bowtie2 version 2.2.9, local;mapping rate: 98.14%
"NS534_HAT1KD_48h_H2AZ_ChIP_4_R1.fq.bz2; NS534_HAT1KD_48h_H2AZ_ChIP_4_R2.fq.bz2";Illumina NextSeq 500, paired-end, 2x76 bp;sequenced reads: 3015430;Bowtie2 version 2.2.9, local;mapping rate: 97.86%
"NS535_HAT1KD_0h_H2AZ_Input_3_R1.fq.bz2; NS535_HAT1KD_0h_H2AZ_Input_3_R2.fq.bz2";Illumina NextSeq 500, paired-end, 2x76 bp;sequenced reads: 3587464;Bowtie2 version 2.2.9, local;mapping rate: 97.15%
"NS536_HAT1KD_0h_H2AZ_Input_4_R1.fq.bz2; NS536_HAT1KD_0h_H2AZ_Input_4_R2.fq.bz2";Illumina NextSeq 500, paired-end, 2x76 bp;sequenced reads: 3176148;Bowtie2 version 2.2.9, local;mapping rate: 97.31%
"NS537_HAT1KD_48h_H2AZ_Input_3_R1.fq.bz2; NS537_HAT1KD_48h_H2AZ_Input_3_R2.fq.bz2";Illumina NextSeq 500, paired-end, 2x76 bp;sequenced reads: 3429705;Bowtie2 version 2.2.9, local;mapping rate: 97.86%
"NS538_HAT1KD_48h_H2AZ_Input_4_R1.fq.bz2; NS538_HAT1KD_48h_H2AZ_Input_4_R2.fq.bz2";Illumina NextSeq 500, paired-end, 2x76 bp;sequenced reads: 2808329;Bowtie2 version 2.2.9, local;mapping rate: 96.6%
"NS539_HAT2KD_0h_H2AZ_ChIP_3_R1.fq.bz2; NS539_HAT2KD_0h_H2AZ_ChIP_3_R2.fq.bz2 ";Illumina NextSeq 500, paired-end, 2x76 bp;sequenced reads: 2060120;Bowtie2 version 2.2.9, local; mapping rate: 95.11%
"NS540_HAT2KD_0h_H2AZ_ChIP_4_R1.fq.bz2; NS540_HAT2KD_0h_H2AZ_ChIP_4_R2.fq.bz2";Illumina NextSeq 500, paired-end, 2x76 bp;sequenced reads: 3065918;Bowtie2 version 2.2.9, local;mapping rate: 95.45%
"NS541_HAT2KD_48h_H2AZ_ChIP_3_R1.fq.bz2; NS541_HAT2KD_48h_H2AZ_ChIP_3_R2.fq.bz2";Illumina NextSeq 500, paired-end, 2x76 bp;sequenced reads: 3308527;Bowtie2 version 2.2.9, local;mapping rate: 98.65%
"NS542_HAT2KD_48h_H2AZ_ChIP_4_R1.fq.bz2; NS542_HAT2KD_48h_H2AZ_ChIP_4_R2.fq.bz2";Illumina NextSeq 500, paired-end, 2x76 bp;sequenced reads: 3561204;Bowtie2 version 2.2.9, local;mapping rate: 98.5%
"NS543_HAT2KD_0h_H2AZ_Input_3_R1.fq.bz2; NS543_HAT2KD_0h_H2AZ_Input_3_R2.fq.bz2";Illumina NextSeq 500, paired-end, 2x76 bp;sequenced reads: 2884766;Bowtie2 version 2.2.9, local;mapping rate: 94.38%
"NS544_HAT2KD_0h_H2AZ_Input_4_R1.fq.bz2; NS544_HAT2KD_0h_H2AZ_Input_4_R2.fq.bz2";Illumina NextSeq 500, paired-end, 2x76 bp;sequenced reads: 2783881;Bowtie2 version 2.2.9, local;mapping rate: 95.24%
"NS545_HAT2KD_48h_H2AZ_Input_3_R1.fq.bz2; NS545_HAT2KD_48h_H2AZ_Input_3_R2.fq.bz2";Illumina NextSeq 500, paired-end, 2x76 bp;sequenced reads: 2961602;Bowtie2 version 2.2.9, local;mapping rate: 97.52%
"NS546_HAT2KD_48h_H2AZ_Input_4_R1.fq.bz2; NS546_HAT2KD_48h_H2AZ_Input_4_R2.fq.bz2";Illumina NextSeq 500, paired-end, 2x76 bp;sequenced reads: 2643620;Bowtie2 version 2.2.9, local;mapping rate: 95.74%
"NS470_BF_2T1_TyRPB9_ChIP_1_R1.fq.gz; NS470_BF_2T1_TyRPB9_ChIP_1_R2.fq.gz";Illumina NextSeq 500, paired-end, 2x76 bp;sequenced reads: 13626619;Bowtie2 version 2.2.9, local;mapping rate: 95.48%
"NS471_BF_2T1_TyRPB9_Input_1_R1.fq.gz; NS471_BF_2T1_TyRPB9_Input_1_R2.fq.gz";Illumina NextSeq 500, paired-end, 2x76 bp;sequenced reads: 12737249;Bowtie2 version 2.2.9, local;mapping rate: 95.55%
"NS474_BF_2T1_TyRPB9_ChIP_2_R1.fq.gz; NS474_BF_2T1_TyRPB9_ChIP_2_R2.fq.gz";Illumina NextSeq 500, paired-end, 2x76 bp;sequenced reads: 12737249;Bowtie2 version 2.2.9, local;mapping rate: 94.08%
"NS475_BF_2T1_TyRPB9_Input_2_R1.fq.gz; NS475_BF_2T1_TyRPB9_Input_2_R2.fq.gz";Illumina NextSeq 500, paired-end, 2x76 bp;sequenced reads: 11622367;Bowtie2 version 2.2.9, local;mapping rate: 94.25%
"NS512_HAT1_KD_TyRPB9_0h_Input_1_R1.fq.bz2; NS512_HAT1_KD_TyRPB9_0h_Input_1_R2.fq.bz2";Illumina NextSeq 500, paired-end, 2x76 bp;sequenced reads: 6396887;Bowtie2 version 2.2.9, local;mapping rate: 97.72%
"NS513_HAT1_KD_TyRPB9_24h_Input_1_R1.fq.bz2; NS513_HAT1_KD_TyRPB9_24h_Input_1_R2.fq.bz2";Illumina NextSeq 500, paired-end, 2x76 bp;sequenced reads: 8439618;Bowtie2 version 2.2.9, local;mapping rate: 97.68%
"NS514_HAT1_KD_TyRPB9_48h_Input_1_R1.fq.bz2; NS514_HAT1_KD_TyRPB9_48h_Input_1_R2.fq.bz2";Illumina NextSeq 500, paired-end, 2x76 bp;sequenced reads: 8144610;Bowtie2 version 2.2.9, local;mapping rate: 96.54%
"NS515_HAT2_KD_TyRPB9_0h_Input_1_R1.fq.bz2; NS515_HAT2_KD_TyRPB9_0h_Input_1_R2.fq.bz2";Illumina NextSeq 500, paired-end, 2x76 bp;sequenced reads: 7919934;Bowtie2 version 2.2.9, local;mapping rate: 97.7%
"NS516_HAT2_KD_TyRPB9_24h_Input_1_R1.fq.bz2; NS516_HAT2_KD_TyRPB9_24h_Input_1_R2.fq.bz2";Illumina NextSeq 500, paired-end, 2x76 bp;sequenced reads: 7887257;Bowtie2 version 2.2.9, local;mapping rate: 98.03%
"NS517_HAT2_KD_TyRPB9_48h_Input_1_R1.fq.bz2; NS517_HAT2_KD_TyRPB9_48h_Input_1_R2.fq.bz2";Illumina NextSeq 500, paired-end, 2x76 bp;sequenced reads: 4348814;Bowtie2 version 2.2.9, local;mapping rate: 97.4%
"NS518_HAT1_KD_TyRPB9_0h_ChIP_1_R1.fq.bz2; NS518_HAT1_KD_TyRPB9_0h_ChIP_1_R2.fq.bz2";Illumina NextSeq 500, paired-end, 2x76 bp;sequenced reads: 9739561;Bowtie2 version 2.2.9, local;mapping rate: 95.54%
"NS519_HAT1_KD_TyRPB9_24h_ChIP_1_R1.fq.bz2; NS519_HAT1_KD_TyRPB9_24h_ChIP_1_R2.fq.bz2";Illumina NextSeq 500, paired-end, 2x76 bp;sequenced reads: 9440267;Bowtie2 version 2.2.9, local;mapping rate: 97.55%

"NS520_HAT1_KD_TyRPB9_48h_ChIP_1_R1.fq.bz2; NS520_HAT1_KD_TyRPB9_48h_ChIP_1_R2.fq.bz2";Illumina NextSeq 500, paired-end, 2x76 bp;sequenced reads: 8647732;Bowtie2 version 2.2.9, local;mapping rate: 93.72%
"NS521_HAT2_KD_TyRPB9_0h_ChIP_1_R1.fq.bz2; NS521_HAT2_KD_TyRPB9_0h_ChIP_1_R2.fq.bz2";Illumina NextSeq 500, paired-end, 2x76 bp;sequenced reads: 11248028;Bowtie2 version 2.2.9, local;mapping rate: 90.78%
"NS522_HAT2_KD_TyRPB9_24h_ChIP_1_R1.fq.bz2; NS522_HAT2_KD_TyRPB9_24h_ChIP_1_R2.fq.bz2";Illumina NextSeq 500, paired-end, 2x76 bp;sequenced reads: 9259488;Bowtie2 version 2.2.9, local;mapping rate: 97.02%
"NS523_HAT2_KD_TyRPB9_48h_ChIP_1_R1.fq.bz2; NS523_HAT2_KD_TyRPB9_48h_ChIP_1_R2.fq.bz2";Illumina NextSeq 500, paired-end, 2x76 bp;sequenced reads: 7396188;Bowtie2 version 2.2.9, local;mapping rate: 91.75%
"NS567_2T1_TyRPB9_HAT1KD_0h_ChIP_2_R1.fq.bz2; NS567_2T1_TyRPB9_HAT1KD_0h_ChIP_2_R2.fq.bz2";Illumina NextSeq 500, paired-end, 2x76 bp;sequenced reads: 4848659;Bowtie2 version 2.2.9, local;mapping rate: 12.33%
"NS568_2T1_TyRPB9_HAT1KD_24h_ChIP_2_R1.fq.bz2; NS568_2T1_TyRPB9_HAT1KD_24h_ChIP_2_R2.fq.bz2";Illumina NextSeq 500, paired-end, 2x76 bp;sequenced reads: 1494552;Bowtie2 version 2.2.9, local;mapping rate: 12.32%
"NS569_2T1_TyRPB9_HAT1KD_48h_ChIP_2_R1.fq.bz2; NS569_2T1_TyRPB9_HAT1KD_48h_ChIP_2_R2.fq.bz2";Illumina NextSeq 500, paired-end, 2x76 bp;sequenced reads: 3314592;Bowtie2 version 2.2.9, local;mapping rate: 6.83%
"NS570_2T1_TyRPB9_HAT1KD_0h_Input_2_R1.fq.bz2; NS570_2T1_TyRPB9_HAT1KD_0h_Input_2_R2.fq.bz2";Illumina NextSeq 500, paired-end, 2x76 bp;sequenced reads: 2378211;Bowtie2 version 2.2.9, local;mapping rate: 85.8%
"NS571_2T1_TyRPB9_HAT1KD_24h_Input_2_R1.fq.bz2; NS571_2T1_TyRPB9_HAT1KD_24h_Input_2_R2.fq.bz2";Illumina NextSeq 500, paired-end, 2x76 bp;sequenced reads: 2024278;Bowtie2 version 2.2.9, local;mapping rate: 83.29%
"NS572_2T1_TyRPB9_HAT1KD_48h_Input_2_R1.fq.bz2; NS572_2T1_TyRPB9_HAT1KD_48h_Input_2_R2.fq.bz2";Illumina NextSeq 500, paired-end, 2x76 bp;sequenced reads: 2469782;Bowtie2 version 2.2.9, local;mapping rate: 87.63%
"NS573_2T1_TyRPB9_HAT2KD_0h_ChIP_2_R1.fq.bz2; NS573_2T1_TyRPB9_HAT2KD_0h_ChIP_2_R2.fq.bz2";Illumina NextSeq 500, paired-end, 2x76 bp;sequenced reads: 3295209;Bowtie2 version 2.2.9, local;mapping rate: 15.08%
"NS574_2T1_TyRPB9_HAT2KD_24h_ChIP_2_R1.fq.bz2; NS574_2T1_TyRPB9_HAT2KD_24h_ChIP_2_R2.fq.bz2";Illumina NextSeq 500, paired-end, 2x76 bp;sequenced reads: 3306559;Bowtie2 version 2.2.9, local;mapping rate: 8.75%
"NS575_2T1_TyRPB9_HAT2KD_48h_ChIP_2_R1.fq.bz2; NS575_2T1_TyRPB9_HAT2KD_48h_ChIP_2_R2.fq.bz2";Illumina NextSeq 500, paired-end, 2x76 bp;sequenced reads: 8180258;Bowtie2 version 2.2.9, local;mapping rate: 7.88%
"NS578_2T1_TyRPB9_HAT2KD_0h_Input_2_R1.fq.bz2; NS578_2T1_TyRPB9_HAT2KD_0h_Input_2_R2.fq.bz2";Illumina NextSeq 500, paired-end, 2x76 bp;sequenced reads: 2331259;Bowtie2 version 2.2.9, local;mapping rate: 84.82%
"NS579_2T1_TyRPB9_HAT2KD_24h_Input_2_R1.fq.bz2; NS579_2T1_TyRPB9_HAT2KD_24h_Input_2_R2.fq.bz2";Illumina NextSeq 500, paired-end, 2x76 bp;sequenced reads: 3043255;Bowtie2 version 2.2.9, local;mapping rate: 86%
"NS580_2T1_TyRPB9_HAT2KD_48h_Input_2_R1.fq.bz2; NS580_2T1_TyRPB9_HAT2KD_48h_Input_2_R2.fq.bz2";Illumina NextSeq 500, paired-end, 2x76 bp;sequenced reads: 3042335;Bowtie2 version 2.2.9, local;mapping rate: 91.39%
"NS563_SM_TyH2A_ChIP_1_R1.fq.bz2; NS563_SM_TyH2A_ChIP_1_R2.fq.bz2";Illumina NextSeq 500, paired-end, 2x76 bp;sequenced reads: 3867340;Bowtie2 version 2.2.9, local;mapping rate: 98.15%
"NS564_SM_TyH2A_ChIP_2_R1.fq.bz2; NS564_SM_TyH2A_ChIP_2_R2.fq.bz2";Illumina NextSeq 500, paired-end, 2x76 bp;sequenced reads: 2877590;Bowtie2 version 2.2.9, local;mapping rate: 98.08%
"NS565_SM_TyH2A_Input_1_R1.fq.bz2; NS565_SM_TyH2A_Input_1_R2.fq.bz2";Illumina NextSeq 500, paired-end, 2x76 bp;sequenced reads: 3189383;Bowtie2 version 2.2.9, local;mapping rate: 96.89%
"NS566_SM_TyH2A_Input_2_R1.fq.bz2; NS566_SM_TyH2A_Input_2_R2.fq.bz2";Illumina NextSeq 500, paired-end, 2x76 bp; sequenced reads: 1159079;Bowtie2 version 2.2.9, local; mapping rate:94.65%

Antibodies

BB2 antibody
Bastin, P., Bagherzadeh, A., Matthews, K. R. & Gull, K. A novel epitope tag system to study protein targeting and organelle biogenesis in Trypanosoma brucei. Mol. Biochem. Parasitol. 77, 235-239 (1996).

H2A.Z antibody
Wedel, C., Förstner, K. U., Derr, R. & Siegel, T. N. GT-rich promoters can drive RNA pol II transcription and deposition of H2A.Z in African trypanosomes. EMBO J 36, 2581-2594 (2017).

Peak calling parameters

For all datasets, sequencing reads were mapped using Bowtie2 local, converted to BAM files using samtools-1.8 view –b, sorted using samtools-1.8 sort and indexed using samtools index. Coverage plots were generated using COVERnant version 0.3.2 (https://github.com/bgbrink/COVERnant) and visualized using GraphPad Prism version 7.0c.

Data quality

FDR5% were not determined as no peak calling was perfomed in this study.

Software

Bowtie 2 (version 2.2.9): Langmead, B. & Salzberg, SL. Fast gapped-read alignment with Bowtie 2. Nat Methods 9(4):357-9 (2012)

SAMtools (version 1.8): Li, H. et al. The Sequence Alignment/Map format and SAMtools. Bioinformatics 25(16):2078-9. (2009)

Python (version 3.6): van Rossum, G. & de Boer, J. Interactively Testing Remote Servers Using the Python Programming Language. CWI Quarterly 4(4):283-303. (1991)

COVERnant (version 0.3.2): https://github.com/bgbrink/COVERnant

GraphPad Prism version 7.0c (GraphPad Software, USA)
