# Supplementary material for: Distinct roles for H4 and H2A.Z acetylation in RNA transcription in African trypanosomes
Source: Nat Commun. 2020 Mar 20;11:1498. doi: 10.1038/s41467-020-15274-0 (PMC7083915; doi:10.1038/s41467-020-15274-0)
Supplement: Supplementary file 4 — Description of Additional Supplementary Files [file 41467_2020_15274_MOESM4_ESM.pdf]

Description of Additional Supplementary Files

File Name: Supplementary Data 1
Description: Overview of T. brucei histone modifications

File Name: Supplementary Data 2
Description: Expression data after HAT1 or HAT2 depletion based on RNAseq

File Name: Supplementary Data 3
Description: Quantification of H2A.Z levels after HAT2 depletion based on western blots

File Name: Supplementary Data 4
Description: Quantification of RNA Pol levels after HAT1 depletion based on western blots

File Name: Supplementary Data 5
Description: ERCC spike in values based on RNA-seq

File Name: Supplementary Data 6
Description: Information on sequencing data used for the individual Figures

File Name: Supplementary Data 7
Description: Statistical analyses of changes in histone acetylation levels
